# Saccharin-Enabled Oral Formulation, Biopharmaceutics and Early Genotoxicity Assessment of the PPARγ Antagonist T0070907

**DOI:** 10.3390/pharmaceutics18101258

**Published:** 2026-10-05

**Authors:** Charlotte Devant, François Xavier Legrand, Maria Eugenia Marques Da Costa, Lilia Sehad, Nathalie Gaspar, Antonin Marchais, Maxime Annereau, Bernard Do

**Affiliations:** 1 Département de Pharmacie Clinique, Institut Gustave Roussy, 114 rue Edouard Vaillant, 94805 Villejuif, France; charlotte.devant@gustaveroussy.fr (C.D.); bernard.do@gustaveroussy.fr (B.D.); 2 CNRS, Institut Galien Paris-Saclay, Université Paris-Saclay, 91400 Orsay, France; francois-xavier.legrand@universite-paris-saclay.fr; 3 BiiOSTeam, INSERM U1360 Biology for Therapeutics in Resistant Pediatric Cancers, Gustave Roussy, Université Paris-Saclay, 94805 Villejuif, France; jenny.marquescosta@gustaveroussy.fr (M.E.M.D.C.); nathalie.gaspar@gustaveroussy.fr (N.G.); antonin.marchais@gustaveroussy.fr (A.M.); 4 Département de Cancérologie de l’Enfant et de l’Adolescent, Institut Gustave Roussy, 114 rue Edouard Vaillant, 94805 Villejuif, France; 5 CNRS, Institut des Sciences Moléculaires d’Orsay, Université Paris-Saclay, 91405 Orsay, France

**Keywords:** T0070907, PPARγ antagonist, saccharin, oral formulation, biopharmaceutics, genotoxicity, developability assessment

## Abstract

**Background/Objectives:** T0070907 is a potent PPARγ antagonist with emerging antitumour activity in osteosarcoma; however, its very low aqueous solubility, uncertain systemic exposure, and nitroaromatic structure raise significant developability concerns. This work aimed to convert T0070907 into a pharmaceutically tractable oral entity and generate an integrated early developability and genotoxicity snapshot. **Methods:** The physicochemical properties of T0070907 were characterised, solubility-enhancing strategies were evaluated, and saccharin was identified as a supramolecular excipient enabling high-dose oral delivery. A 20 mg/mL oral liquid formulation was developed and monitored using a validated, stability-indicating UHPLC/LC–MS method. In vivo systemic and tissue exposure was assessed in rats using LC–MS/MS; hepatic metabolism was explored in human liver microsomes; and genotoxicity risk was evaluated both in silico (ICH M7) and in a GLP-compliant Ames test. **Results:** Saccharin markedly enhanced solubility and enabled a 20 mg/mL oral formulation that remained stable for 90 days, with pyridin-4-amine identified as the dominant degradation product. In rats, T0070907 was below the limit of quantification in plasma after oral dosing at 5, 10 and 100 mg/kg, including in a solubilising vehicle. In contrast, T0070907 was clearly mutagenic in the Ames test. **Conclusions:** These findings support saccharin-based supramolecular formulations as a practical strategy for poorly soluble nitrobenzamides, while positioning T0070907 primarily as a pharmacological tool.

## 1. Introduction

Osteosarcoma is the most common primary malignant bone tumour in adolescents and young adults [1]. Despite intensification of surgery and systemic therapy, outcomes have plateaued: 5-year overall survival is ~60–70% for localised disease but falls to 10–20% in metastatic or relapsed settings, with most patients treated with high-dose methotrexate, doxorubicin and cisplatin (MAP) plus surgery [2]. Late relapses and treatment-related toxicities remain major concerns, and there is still no validated targeted agent that consistently improves survival in high-risk osteosarcoma. This has motivated interest in repurposing or redirecting small molecules that modulate signalling pathways relevant to osteoblast differentiation, bone remodelling and tumour growth.

Among these pathways, nuclear receptors, including retinoic acid receptor β (RARβ), peroxisome proliferator-activated receptor γ (PPARγ), liver X receptors (LXRs) and Rev-Erbα, have recently emerged as attractive targets in osteosarcoma. In a large chemical biology screen, selective modulators of these receptors, including PPARγ antagonists, inhibited osteosarcoma cell growth in vitro and in vivo, at least in part via mTOR pathway suppression. PPARγ also regulates osteoblastogenesis and bone marrow stromal cell differentiation, and its transcriptional network can be rewired in cancer and stem-cell contexts [3]. These observations provide a mechanistic rationale for exploring PPARγ antagonism as a therapeutic strategy in osteosarcoma.

T0070907 is an irreversible PPARγ antagonist/inverse agonist that binds the ligand-binding domain and blocks ligand-induced activation. Beyond its role as a biochemical probe, T0070907 has demonstrated antiproliferative and pro-apoptotic effects in several tumour models, including colorectal and breast cancer, often accompanied by cell-cycle arrest and reduced motility [4,5]. More recently, T0070907 has been highlighted as a small molecule of interest in osteosarcoma progression, in part through modulation of PPARγ-dependent transcriptional programmes [3]. However, almost all published work has treated T0070907 as a “tool compound”: typically dissolved in high proportions of organic solvent (e.g., DMSO) for in vitro assays, with limited or no information on its physicochemical behaviour, formulation, systemic exposure or toxicological liabilities.

From a pharmaceutical perspective, T0070907 poses several challenges typical of late-discovery nuclear receptor ligands. It is a highly crystalline, poorly water-soluble nitrobenzamide with limited solubility in pharmaceutically acceptable vehicles, placing it in a high-risk developability space where limited apparent solubility and precipitation are expected to compromise systemic exposure. To enable robust preclinical dosing and an early developability snapshot, formulation feasibility and genotoxicity risk must be assessed in parallel (ICH M7 framework).

Solubility-enabling strategies for such compounds usually rely on co-solvency, surfactants, lipid-based systems, amorphous dispersions, or cyclodextrin complexes, and classical approaches were therefore screened. Another solubilisation approach relies on specific hydrogen-bond donor/acceptor interactions to form drug–excipient association states that can increase apparent solubility and mitigate precipitation. Among excipients with these properties, saccharin (E954) is a long-standing oral sweetener and pharmacopeial excipient with a well-characterised safety profile and extensive use in taste masking. Beyond its organoleptic role, saccharin has been used as a supramolecular partner capable of engaging in strong, directional interactions with multiple APIs, sometimes as salts or cocrystals [6,7,8,9,10]. However, its use as a functional solubilising excipient for neutral or weakly acidic, poorly soluble nitrobenzamide ligands such as T0070907, particularly for high-strength oral liquids, remains underexplored.

In this study, the objective was to convert T0070907 from an insoluble compound into a pharmaceutically tractable entity and to generate an integrated early developability snapshot. First, physicochemical characterisation and screening of conventional solubilisation approaches established the key developability properties of T0070907. A saccharin-based strategy markedly increased T0070907 solubility, and the resulting solid-state systems were characterised by FTIR microscopy and powder X-ray diffraction (PXRD). The resulting 20 mg/mL oral liquid formulation underwent stability assessment and stress testing using a stability-indicating UHPLC/LC–MS method, which also enabled monitoring of the dominant degradation product. Finally, metabolic stability was assessed in human liver microsomes, while genotoxicity was evaluated through an ICH M7–aligned in silico assessment followed by a GLP-compliant Ames test.

The present study establishes a pharmaceutical and safety framework for T0070907, including a formulation suitable for high-dose oral administration and a first assessment of its metabolic and genotoxicity profiles. Antitumour efficacy, for which osteosarcoma provides the therapeutic context, was not addressed at this stage. The successful formulation of T0070907 demonstrates that its poor aqueous solubility can be overcome, whereas the very low systemic exposure and positive Ames test identify important limitations that will require further investigation. These findings provide a basis for defining the potential and boundaries of T0070907 for future pharmacological and preclinical studies.

## 2. Materials and Methods

### 2.1. Chemicals and Reagents

T0070907 (batch 434289; purity > 99%) and GW9662 (internal standard for LC–MS quantification) were purchased from MedChemExpress (Monmouth Junction, NJ, USA). Acetonitrile (UHPLC grade), hydrogen peroxide (H_2_O_2_, 30% *v*/*v*), hydrochloric acid (HCl), sodium hydroxide (NaOH), orthophosphoric acid, polyethylene glycol (PEG) 400 and 600, propylene glycol (PG), saccharin (base), polyvinylpyrrolidone K10 and K30 (PVP K10 and K30), Kolliphor^®^ EL, Tween^®^ 20 and Tween^®^ 60, sucralose and other analytical-grade reagents were obtained from Sigma-Aldrich (St Quentin-Fallavier, France). 4-Aminopyridine (≥99% purity) was purchased from Sigma-Aldrich (Saint-Quentin-Fallavier, France) and used as an authentic reference standard for the identification of DP1. Labrafil^®^ M 1944 CS, Labrasol^®^ ALF and Transcutol^®^ HP were obtained from Gattefossé (Saint-Priest, France). Hydroxypropyl-β-cyclodextrin (HP-β-CD, Roquette, France), hydroxypropyl-γ-cyclodextrin (HP-γ-CD, Ashland, Wilmington, DE, USA) and sulfobutylether-β-cyclodextrin (SBE-β-CD, Ligand, Jupiter, FL, USA) were used as received. Dimyristoylphosphatidylcholine (DMPC) and dipalmitoylphosphatidylcholine (DPPC) were purchased from Avanti Polar Lipids (Alabaster, AL, USA).

Gelatin capsules were obtained from Cooper (Melun, France), vegetable capsules from LGA (La Seyne-sur-Mer, France) and hard HPMC capsules (Licaps^®^ and Vcaps Plus^®^) from Lonza (Colmar, France). Peppermint flavours and a cooling agent were supplied by IFF (New York, NY, USA). Human liver microsomes (HLMs, batch MIC259936) were purchased from Biopredic (Saint-Grégoire, France). Water was purified by reverse osmosis and further filtered (Milli-Q^®^ system, Millipore, Molsheim, France).

### 2.2. Physicochemical Characterisation of T0070907

#### 2.2.1. Solubility

Equilibrium solubility of T0070907 was determined by the shake-flask method. An excess amount of T0070907 was accurately weighed into glass vials (*n* = 3 per condition), and 1 mL of test solvent was added (water, buffer, co-solvents or selected formulation vehicles). Vials were shaken at room temperature (22–25 °C) for 24 h. The presence of undissolved solid was checked visually before sampling. Suspensions were either centrifuged at 10,000 rpm for 2 min or filtered through 0.22 µm filters, and the resulting supernatants or filtrates were diluted as appropriate and analysed by UHPLC. Solubility was expressed as the mean concentration ± standard deviation.

#### 2.2.2. Distribution Coefficient (log D)

The apparent distribution coefficient at pH 7.4 (log D_7.4_) was determined by a shake-flask method in 1-octanol/HEPES buffer. Ten microlitres of a 10 mM T0070907 stock solution in DMSO were added to 490 µL of a 1:1 (*v*/*v*) pre-saturated octanol/HEPES buffer mixture (pH 7.4). The biphasic system was gently shaken at room temperature for 2 h, then allowed to stand until phase separation. Aliquots (10 µL) from each phase were diluted in methanol (490 µL) and analysed by LC–MS. log D_7.4_ was calculated as log(C_octanol_/C_buffer_). Experiments were performed in triplicate.

#### 2.2.3. pKa Determination

The ionisation constant of T0070907 was determined by pH-metric titration in a methanol/water mixture. T0070907 was dissolved in a methanol/water solution containing a known amount of strong acid. Titrations were performed at 25 °C using an Omnis titrator (Metrohm, Herisau, Switzerland) equipped with a combined glass microelectrode calibrated at pH 4.0, 7.0 and 10.0. A 0.100 M NaOH solution in methanol/water was used as titrant. pH–volume curves were analysed with Hyperquad software 2013 to obtain acidity constants. Aqueous pKa values were extrapolated from mixed-solvent data based on the dielectric constant and water molarity.

#### 2.2.4. Thermal Analysis

Thermogravimetric analysis (TGA) was carried out on T0070907 using an STA 6000 thermogravimetric analyser (PerkinElmer, Waltham, MA, USA). Approximately 13–14 mg of sample was weighed into open alumina crucibles. After an initial equilibration at 30 °C, samples were heated to 900 °C at 10 °C/min under nitrogen (20 mL/min), followed by a 10 min isothermal step at 900 °C under air.

High-speed differential scanning calorimetry (hyperDSC^®^) was performed on a DSC 8500 (PerkinElmer) to determine the melting behaviour of T0070907. Approximately 1 mg of sample was sealed in aluminium pans. After an isothermal hold at −80 °C for 1 min, the temperature was ramped from −80 to 280 °C at 700 °C/min under nitrogen (20 mL/min).

### 2.3. Solubilisation Strategies

#### 2.3.1. Co-Solvency

The solubility of T0070907 in individual co-solvents (PEG 400, PEG 600, PG, Transcutol^®^ HP (Gattefosse, St Pirest, France) and selected mixtures with water) was determined as described in Section 2.2.1. The best-performing co-solvents were further considered for formulation development.

#### 2.3.2. Cyclodextrin Inclusion

Inclusion complexation with HP-β-CD, HP-γ-CD and SBE-β-CD was investigated. T0070907 was added in excess to aqueous cyclodextrin solutions at various concentrations. Suspensions were stirred for 24 h at room temperature, centrifuged and analysed by UHPLC to quantify dissolved T0070907.

#### 2.3.3. Lipid-Based Systems

Lipid-based vehicles and surfactants, including Labrafil^®^ M 1944 CS, Labrasol^®^ ALF, Tween^®^ 20, Tween^®^ 60 and Kolliphor^®^ EL were screened for their ability to solubilise T0070907, as described in Section 2.2.1.

Interactions of T0070907 with DMPC and DPPC were investigated by differential scanning calorimetry (DSC) at three T0070907 molar ratios (5, 10, and 15 mol%), following the sample preparation and DSC procedures previously described [11].

#### 2.3.4. Saccharin-Based Systems and Preparation of T0070907–Saccharin Mixtures

Saccharin was investigated as a functional solubilising excipient. Solid mixtures of T0070907 and saccharin were prepared at molar ratios ranging from 0:3 to 3:0 (T0070907:saccharin) to reach a total mass of 200 mg. Powders were dissolved in 40 mL of methanol, the solvent was removed under a nitrogen stream, and the residue was redispersed in water and freeze-dried. The dried residue was used for FTIR imaging and XRPD analysis.

#### 2.3.5. FTIR Imaging of T0070907–Saccharin Mixtures

Fourier transform infrared (FTIR) imaging was performed using a PerkinElmer MIR/NIR Spotlight 400 imager (PerkinElmer, Waltham, MA, USA). Samples were analysed by attenuated total reflectance (ATR) in the mid-IR region (4000–690 cm^−1^), with a spectral resolution of 8 cm^−1^ and a pixel size of 1.5 µm.

#### 2.3.6. X-Ray Powder Diffraction (XRPD)

X-ray powder diffraction (XRPD) measurements were performed using an Aeris Research Edition diffractometer (Malvern Panalytical, Palaiseau, France) equipped with a six-position sample changer and a PIXcel 1D detector. The instrument was operated at 300 W (30 kV, 10 mA) using Cu Kα radiation (Kα1 + Kα2; λ ≈ 1.5406 Å). Samples were mounted on Si (510) zero-background sample holders (AlpyX, Sainte-Hélène-du-Lac, France). Diffraction patterns were collected over a 2θ range of 2–60°, with a total acquisition time of 10 min per sample and continuous sample rotation during data collection. XRPD analyses were performed on T0070907 and saccharin before and after freeze-drying, as well as on freeze-dried T0070907-saccharin mixtures prepared at saccharin/T0070907 molar ratios of 1:3, 1:2, 1:1, 1.6:1, 2:1 and 3:1.

### 2.4. Oral Solution Formulation

A saccharin-based oral liquid formulation of T0070907 was developed using PEG 400 and PG as co-solvents and PVP as a crystallisation inhibitor. To ensure complete complexation and prevent precipitation, a saccharin/T0070907 molar ratio of 1.6:1 (mass ratio 1:1) was selected. A saccharin base was used, as saccharin salts led to rapid precipitation when combined with T0070907.

For the final formulation (target T0070907 concentration of 20 mg/mL), PG and PEG 400 were weighed and mixed, followed by dissolution of saccharin, which was heated at 70 °C under stirring. PVP K10 and K30 were then added until completely dissolved. Finally, T0070907 was introduced gradually, and the mixture was stirred until a clear, homogeneous solution was obtained. The pH of the formulation was approximately 5.0.

All excipients were used within pharmacopeial limits for oral administration and remained stable at pH 5.0 [12].

For capsule filling trials, T0070907-free placebo solutions with identical excipient composition were used to evaluate capsule integrity and leakage over 90 days at room temperature in amber glass bottles. This capsule trial was performed on placebo solutions only and is distinct from the stability study described in Section 2.6, which was carried out on the T0070907-containing solution stored in amber glass vials.

### 2.5. Analytical Methods for Assay and Stability Monitoring

UHPLC analyses were performed on an Agilent 1290 Infinity II system equipped with a UV detector set at 254 nm and an XBridge C18 column (150 × 4.6 mm, 3.5 µm; Waters, Milford, MA, USA) maintained at 45 °C. The mobile phase consisted of 2 mM ammonium formate buffer, pH 3.1 (solvent A) and acetonitrile (solvent B). The gradient programme was: 0–2 min, 95% A; 2–10 min, 95–5% A; and 10–15 min, 5% A. The flow rate was 1.0 mL/min and the injection volume was 1 µL.

For LC–MS analyses, the UHPLC system was coupled to an Agilent 6495 triple quadrupole mass spectrometer. The same column and flow rate were used. A binary gradient of water with 1% formic acid (solvent A) and acetonitrile with 1% formic acid (solvent B) was applied, ramping from 5% to 95% B over 2.4 min and maintaining 95% B until 4.0 min. Data were acquired and processed using MassHunter Qualitative Analysis 10.0 (Agilent, Santa Clara, CA, USA).

Multiple reaction monitoring (MRM) was used for quantification. For T0070907, the precursor ion *m*/*z* 278.1 was monitored with product ions at *m*/*z* 74, 75, 96, 110 and 138. GW9662 was monitored with an analogous transition set. Source parameters were optimised by direct infusion of standards.

The UHPLC and LC–MS methods were validated according to ICH Q2(R2) guidelines for specificity, linearity, accuracy, precision, LOD and LLOQ [13]. Excipients were systematically injected to verify the absence of interference and matrix effects. The in-house LC–MS method was validated for assay and stability monitoring of the formulation and for exploratory analysis of biological samples. With a lower limit of quantification of 109.88 ng/mL in plasma, it is not sensitive enough to support conclusions on systemic exposure; all exposure conclusions in this work therefore rely on the Eurofins LC–MS/MS method (2 ng/mL in plasma, 2 ng/g in tissue homogenates).

### 2.6. Forced Degradation and Stability Studies

Forced degradation was performed in solution following ICH Q1A(R2) to assess the susceptibility of T0070907 to hydrolytic and oxidative conditions [14]. T0070907 was dissolved at 1 mg/mL in 1 M HCl, 1 M NaOH or 3% H_2_O_2_ and incubated at 60 °C in amber glass vials. Samples were withdrawn at 0, 2, 4, 24, 48 and 96 h, neutralised when appropriate and analysed by UHPLC and LC–MS to monitor T0070907 degradation and identify degradation products.

The stability of the saccharin-based oral solution (20 mg/mL T0070907) was evaluated according to ICH Q1A(R2) and SFPC/GERPAC guidelines [14,15]. Three independent 5 mL batches were prepared, filled into amber glass vials and stored at room temperature. At predefined time points (day 0, 7, 14, 21, 28, 35, 42, 60 and 90), samples were inspected visually for colour change, turbidity and visible particles, and T0070907 concentration and pH were measured. Degradation products were monitored by the validated LC–MS method. Acceptance criteria were defined a priori as a T0070907 content within 90–110% of the initial value, absence of visible particles or colour change, and a pH within ±0.5 units of the initial value. The study followed the SFPC/GERPAC guidelines for stability studies of hospital pharmaceutical preparations and was not intended as an ICH Q1A(R2) registration stability programme; protection from light was provided by the container closure system (amber glass vials and opaque hard capsules), and humidity is not a relevant stress factor for a liquid in a closed container.

### 2.7. Animals and In Vivo Dosing

An in-house animal study was conducted in female mice to match the tumour models for which the formulation was developed, housed under standard conditions with ad libitum access to food and water. Animals received T0070907 at 10 mg/kg once daily by oral gavage in the saccharin-based formulation for 20 to 35 consecutive days, at a dose volume of 0.5 mL/kg. A total of 15 animals were used ([6] treated and [6] vehicle controls), allocated to groups through randomisation. Control animals received an equivalent volume of vehicle alone. On the final day of dosing, animals were euthanised 1 h after administration. Blood samples were collected into heparinised tubes and centrifuged to obtain plasma, which was stored at −20 °C until analysis. Hind limb bones were harvested, cleared of soft tissues, frozen at −20 °C, and processed as described below (protocols are detailed in the Supplementary Materials).

To confirm these findings, complementary in vivo studies were performed by Eurofins to evaluate systemic and tissue exposure to the parent compound T0070907, using an LC–MS/MS method adapted from that developed as previously described (LLOQ: 2 ng/mL in plasma; 2 ng/g in tissue homogenates). Two rat studies were performed. In the first study, male Sprague-Dawley rats received T0070907 by oral gavage (5 or 10 mg/kg) or intravenous injection (1 mg/kg) to characterise plasma pharmacokinetics. Serial plasma samples were collected up to 24 h post-dose (oral: 0.167–24 h; IV: 0.05–24 h). Femurs were collected at study termination, homogenised, and analysed by LC–MS/MS.

In the second study, early plasma exposure was assessed following oral dosing at 100 mg/kg or intravenous dosing at 2 mg/kg of T0070907 dissolved in DMSO, with plasma samples collected from 0.05 to 1 h post-dose. At 1 h post-dose, animals were euthanised and tissues (liver, kidney, brain, and thigh muscle) were collected, rinsed with saline, weighed, and homogenised in PBS (pH 7.4) prior to LC–MS/MS analysis. Blood samples were collected into lithium-heparin tubes, centrifuged within 1 h, and plasma and tissue homogenates were stored frozen (≤−70 °C) until analysis.

All animal procedures complied with national regulations on animal experimentation and were approved by the relevant ethics committee (authorisation APAFIS#27183-2020091414229028) and under Eurofins Taiwan Pharmacology Discovery Services Taiwan, Ltd. 25, Wugong 6th Road, Wugu District New Taipei City, 24,891 studies ODS112-01061300 (first rat study) and ODS112-01077544 (second rat study).

### 2.8. Biological Sample Preparation and LC–MS/MS Quantification

#### 2.8.1. Internal Standard and Calibration Standards

A stock solution of GW9662 (1 mg/mL) was prepared in DMSO and stored at −20 °C. Internal standard solutions were freshly prepared in methanol. Calibration standards and quality control (QC) samples were prepared by spiking blank rat plasma with known concentrations of T0070907 and a fixed amount of GW9662.

#### 2.8.2. Plasma Sample Preparation by Solid-Phase Extraction

T0070907 and GW9662 were extracted from plasma using C18 Chromabond^®^ solid-phase extraction cartridges (Macherey-Nagel, Germany). Cartridges were conditioned with 1 mL methanol followed by 1 mL water. Plasma samples (100 µL) were spiked with 10 µL of GW9662 working solution, vortexed and loaded onto the cartridges. After sample loading, cartridges were washed with 2 mL water and dried under vacuum. Analytes were eluted with 1 mL methanol. Eluates were evaporated under a gentle stream of air at 40 °C and reconstituted in mobile phase prior to LC–MS analysis.

#### 2.8.3. Bone Sample Preparation

For each rat, organs were collected, rinsed with saline, gently cleaned of soft tissue and briefly washed with acetone. Bones were dried for 1 h at 50 °C and then manually ground in a mortar. Aliquots of bone powder were weighed into threaded glass tubes.

For control bones, 2 µg of T0070907 reference solution (20 µg/mL in water with 1% formic acid and 0.05% orthophosphoric acid) was added to allow calculation of extraction yield. Samples were incubated in 1 mL methanol at 50 °C for 12 h under gentle agitation. The supernatants were collected and evaporated under a stream of air at 70 °C. Residues were reconstituted in 190 µL water and 10 µL orthophosphoric acid, vortexed and centrifuged, and the clear supernatant was analysed by LC–MS using the method described in Section 2.5.

T0070907 concentrations in bone extracts were corrected for extraction yield, determined from spiked control samples. Results were expressed as ng of T0070907 per mg of dry bone.

### 2.9. Metabolic Stability in Human Liver Microsomes

The in vitro metabolic stability of T0070907 was investigated in pooled human liver microsomes (HLMs). HLMs were used due to their cost-effectiveness, robustness, and suitability for high-throughput applications. They also exhibit low lot-to-lot variability, as they are derived from pooled donors, and are widely available from reputable commercial sources [16]. Incubations (final volume 500 µL) contained T0070907 (10 µM, 1% DMSO), HLM (0.5 mg/mL protein), 100 mM PBS pH 7.4 and an NADPH-regenerating system (1 mM NADPH). Control incubations included (i) T0070907 without microsomes, (ii) microsomes with NADPH but no T0070907 and (iii) a positive control with midazolam (10 µM).

Samples were pre-incubated for 5 min at 37 °C, and reactions were initiated by adding NADPH. At 0, 15, 30, 60 and 90 min, 50 µL aliquots were removed and quenched with 150 µL ice-cold acetonitrile. Precipitated proteins were removed by centrifugation, and supernatants were analysed by LC–MS as described above. Each condition was tested in triplicate in three independent experiments.

### 2.10. In Silico Genotoxicity Assessment

In silico evaluation of mutagenic potential was performed in line with ICH M7 recommendations [17]. The structure of T0070907 was assessed using two complementary software tools: Toxtree 3.0 (rule-based expert system) and the Toxicity Estimation Software Tool 5.1.1 (T.E.S.T., US EPA; QSAR-based) [18,19]. Structures were provided as SMILES strings.

### 2.11. Ames Bacterial Reverse Mutation Test

A bacterial reverse mutation (Ames) test was performed under GLP by the Toxicology Laboratory of Institut Pasteur de Lille (France), in accordance with OECD Test Guideline 471and EU regulation EC 440/2008 [20,21,22]. The test used *Salmonella typhimurium* strains TA1535, TA1537, TA98, TA100 and TA102, maintained in liquid nitrogen at the test facility. TA1535 and TA100 detect base-pair substitution mutations, whereas TA1537 and TA98 detect frameshift mutations. TA102 contains an ochre mutation in the hisG gene and is sensitive to oxidative and cross-linking agents.

A preliminary dose-range finding assay was performed to assess toxicity and solubility under the test conditions. No toxicity was observed, whereas precipitation limited the maximum dose used in the main assays to 1500 µg/plate. T0070907 was subsequently tested at 15, 50, 150, 500, and 1500 µg/plate in two independent assays, both in the absence and presence of metabolic activation (S9 mix), together with appropriate solvent and positive controls. In the second assay, the pre-incubation method was used with metabolic activation for strains TA1535, TA1537, and TA102, whereas the standard plate-incorporation method was retained for TA98 and TA100. Plates were incubated at approximately 37 °C for 48 or 72 h, and revertant colonies were counted. The test item was a T0070907 solution in DMF (92.9 mg/mL). Mutagenicity was assessed based on biologically relevant increases in revertant counts, taking into account the magnitude of the response, the presence of a concentration–response relationship, and comparison with historical control data. The T0070907 used for the Ames test had not undergone prior stress; therefore, the assay primarily assessed the mutagenic potential of the parent compound rather than that of its degradation product DP1. Individual revertant counts and corresponding control data are provided in the Supplementary Data.

### 2.12. Statistical Analysis

Analytical methods (UHPLC and LC–MS) were validated in terms of linearity, accuracy, precision and limits of detection and quantification according to ICH Q2(R2) [13]. Linearity was assessed using a Cochran test followed by ANOVA. Intra-day precision was evaluated by analysing samples in triplicate at six concentration levels (UHPLC) or seven levels (LC–MS) within a single day; inter-day precision was determined by repeating the same analyses on three consecutive days. The lower limit of quantification was set as the lowest concentration giving a signal-to-noise ratio of at least 10 and meeting the ICH Q2(R2) accuracy and precision criteria (relative bias within ± 10%, coefficient of variation ≤ 10%). Accuracy was expressed as relative bias, precision as coefficient of variation (CV) and total error as the sum of relative bias and inter-day precision.

Unless otherwise stated, data are presented as mean ± standard deviation.

## 3. Results

### 3.1. Physicochemical Characterisation

T0070907 is a nitrobenzamide derivative (C_12_H_8_ClN_3_O_3_, 277.6 g/mol) with two ionisable centres: one very weakly acidic (pKa_1_ = 5.4, assigned to the conjugate acid of the pyridine nitrogen, whose basicity is lowered by the electron-withdrawing amide substituent) and one weakly basic (pKa_2_ = 14.2, assigned to the amide N–H). The compound has an apparent distribution coefficient at pH 7.4 (log D_7.4_) of 1.76 (Table 1) and is highly crystalline, with a sharp melting endotherm at 260 °C. Thermogravimetric analysis showed an onset of degradation at 185 °C, with no significant mass loss below this temperature, indicating the absence of volatile solvent or water.

In water, T0070907 was practically insoluble (equilibrium solubility < 0.1 mg/mL). Solubility increased in organic solvents, reaching 5 mg/mL in methanol, 60–90 mg/mL in DMSO and 100–140 mg/mL in DMF (Table 1). These data confirmed that aqueous solubility was far below that required for an oral solution containing 20 mg/mL of T0070907 and motivated the exploration of solubilisation strategies.

Taken together, these properties place T0070907 in a high-risk developability space where low apparent solubility/precipitation is likely to limit exposure for oral absorption.

### 3.2. Solubilisation Strategies

#### 3.2.1. Co-Solvents

The solubility of T0070907 was first assessed in pharmaceutically acceptable co-solvents (Table 2). At room temperature, PEG 400 (15.66 mg/mL), PEG 600 (15.80 mg/mL) and Transcutol^®^ HP (13.56 mg/mL) produced the highest solubility values, whereas propylene glycol alone was less effective (≤4.69 mg/mL). Although these PEG-based co-solvents markedly increased T0070907 solubility compared with water, none achieved the target concentration of 20 mg/mL. Moreover, the high proportions of PEG required would substantially increase formulation viscosity, limiting the suitability of a co-solvent-based approach for the development of a 20 mg/mL oral solution.

#### 3.2.2. Surfactant-Based Systems

The solubility of T0070907 was also assessed in pharmaceutically acceptable surfactant-based vehicles (Table 2). At room temperature, Tween^®^ 60 and Tween^®^ 20 provided the highest apparent solubilities, reaching 15.27 and 12.62 mg/mL, respectively. Labrasol^®^ ALF and Kolliphor^®^ EL showed intermediate solubilising capacities, with apparent solubilities of 11.41 and 8.13 mg/mL, respectively, whereas Labrafil^®^ was markedly less effective (1.07 mg/mL). None of these vehicles achieved the target concentration of 20 mg/mL, and attempts to prepare formulations at this concentration resulted in heterogeneous systems, with visible precipitation or phase separation upon standing. Therefore, surfactant-based solubilisation was not considered a suitable strategy and was not pursued further.

#### 3.2.3. Cyclodextrin Inclusion

Complexation with three pharmaceutical-grade cyclodextrins (SBE-β-CD, HP-β-CD and HP-γ-CD) was investigated using a fixed 1:5 T0070907:CD molar ratio (Table 3).

Solubility of T0070907 in these aqueous CD solutions remained low (1.13–1.47 mg/mL), representing only a modest increase over pure water. Under the conditions tested, there was no evidence of efficient inclusion complex formation that could justify using cyclodextrins as primary solubilising agents for this compound.

#### 3.2.4. Phospholipid-Based Systems

To investigate whether liposomes could represent a suitable formulation strategy for T0070907, its interaction with saturated phospholipid bilayers was investigated by DSC (Supplementary Figure S1) using DMPC and DPPC, which exhibit a pre-transition (L_β_′ → P_β_′) followed by the main transition to the liquid-crystalline phase (P_β_′ → L_α_). T0070907 was tested at molar ratios of 5, 10 and 15 mol%. For both phospholipids, the main transition temperature remained essentially unchanged, with only minor changes in enthalpy, while the pre-transition remained detectable at all compositions despite moderate changes in temperature and enthalpy. These effects may reflect interactions of the small soluble fraction of T0070907 with the phospholipid headgroups and/or the presence of excess crystalline drug. Overall, the limited effect on the main transition suggests little, if any, incorporation of T0070907 into the hydrophobic region of the bilayers, indicating that liposomes were unlikely to provide a suitable formulation strategy for T0070907.

#### 3.2.5. Saccharin-Based Systems and Solid-State Characterisation

Saccharin, a well-established pharmaceutical excipient, was then evaluated as a supramolecular solubilising excipient. Addition of saccharin to PEG/propylene glycol mixtures led to a marked increase in the apparent solubility of T0070907, enabling clear solutions at concentrations compatible with the target dose.

**Figure 1:**
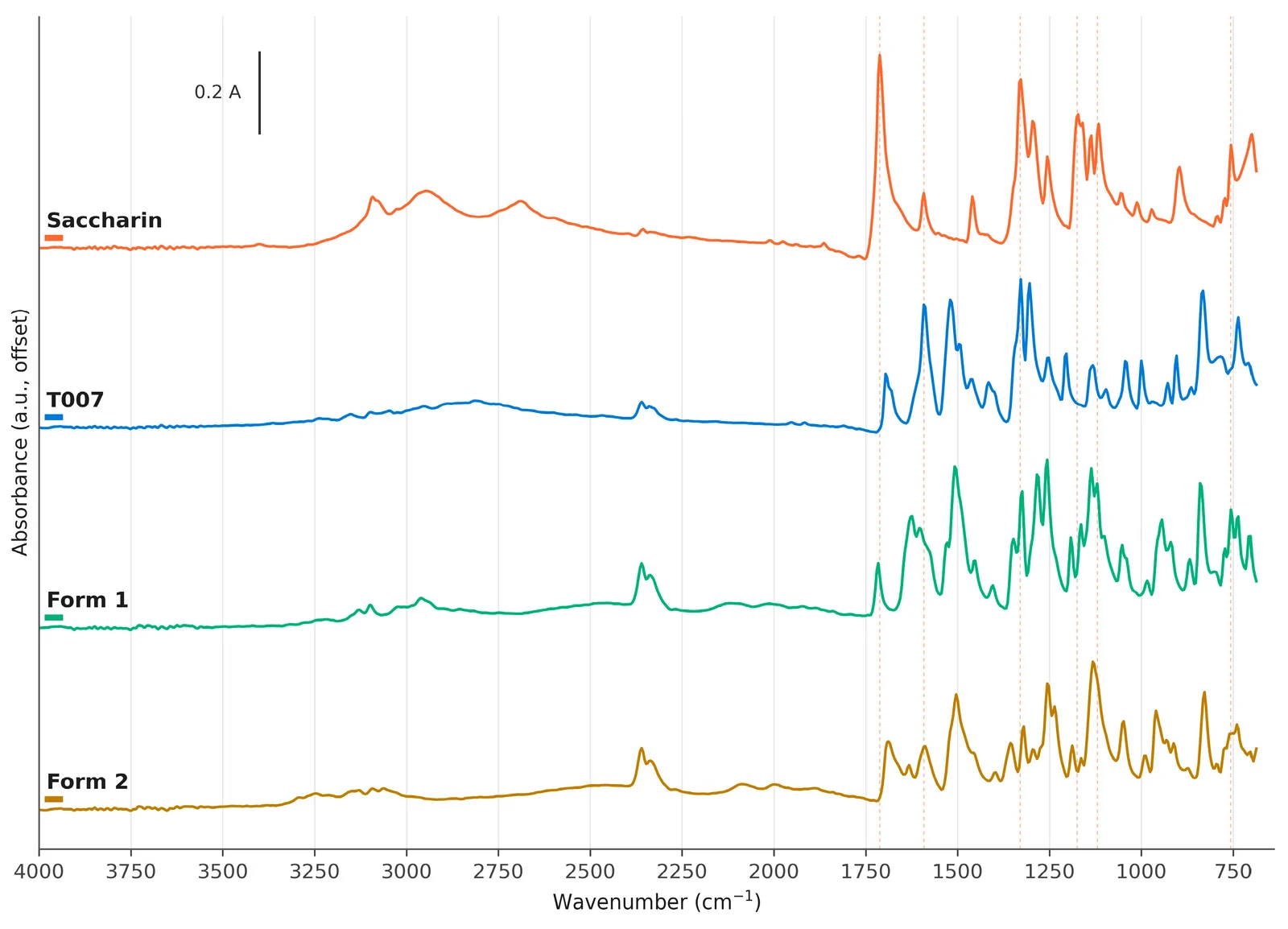
FTIR spectra of T0070907, saccharin and freeze-dried T0070907–saccharin interaction signatures. Overlaid mid-IR spectra of pure T0070907, pure saccharin and two freeze-dried FTIR-defined interaction signatures (F1 and F2), highlighting band (dot point) shifts and intensity changes in characteristic regions consistent with drug–excipient interactions.

To investigate the molecular interactions between T0070907 and saccharin that could contribute to its enhanced solubilisation, freeze-dried binary mixtures of T0070907 and saccharin prepared at various molar ratios were characterised by FTIR imaging. Comparison of FTIR spectra of pure T0070907 and saccharin with those of the two new interaction phases identified by principal component analysis (PCA) of the freeze-dried binary mixtures (FTIR interaction signatures F1 and F2) showed multiple band shifts and intensity changes (Figure 1). Notably, shifts were observed in the nitro stretching region of T0070907 and in the C=O and S=O regions of T0070907 and saccharin. These spectral changes are consistent with strong drug–excipient interactions, plausibly involving hydrogen bonding and/or changes in the local protonation environment. The modifications were more pronounced for signature F2, including in the ~3100 cm^−1^ region associated with the saccharin N–H band, suggesting a stronger interaction state relative to signature F1.

However, because FTIR provides indirect evidence of interaction and was not complemented here by orthogonal solid-state techniques (e.g., XRPD/DSC) capable of unambiguous phase assignment, we refer to these as FTIR interaction signatures F1 and F2 and make no assignment of phase, salt or cocrystal. F1 and F2 were observed to coexist within the same freeze-dried sample, and no combination of molar ratio and lyophilisation conditions was found that yielded either signature alone, so they are not described here as separable phases (characteristic bands used for interpretation are summarised in Table 1).

**Figure 2:**
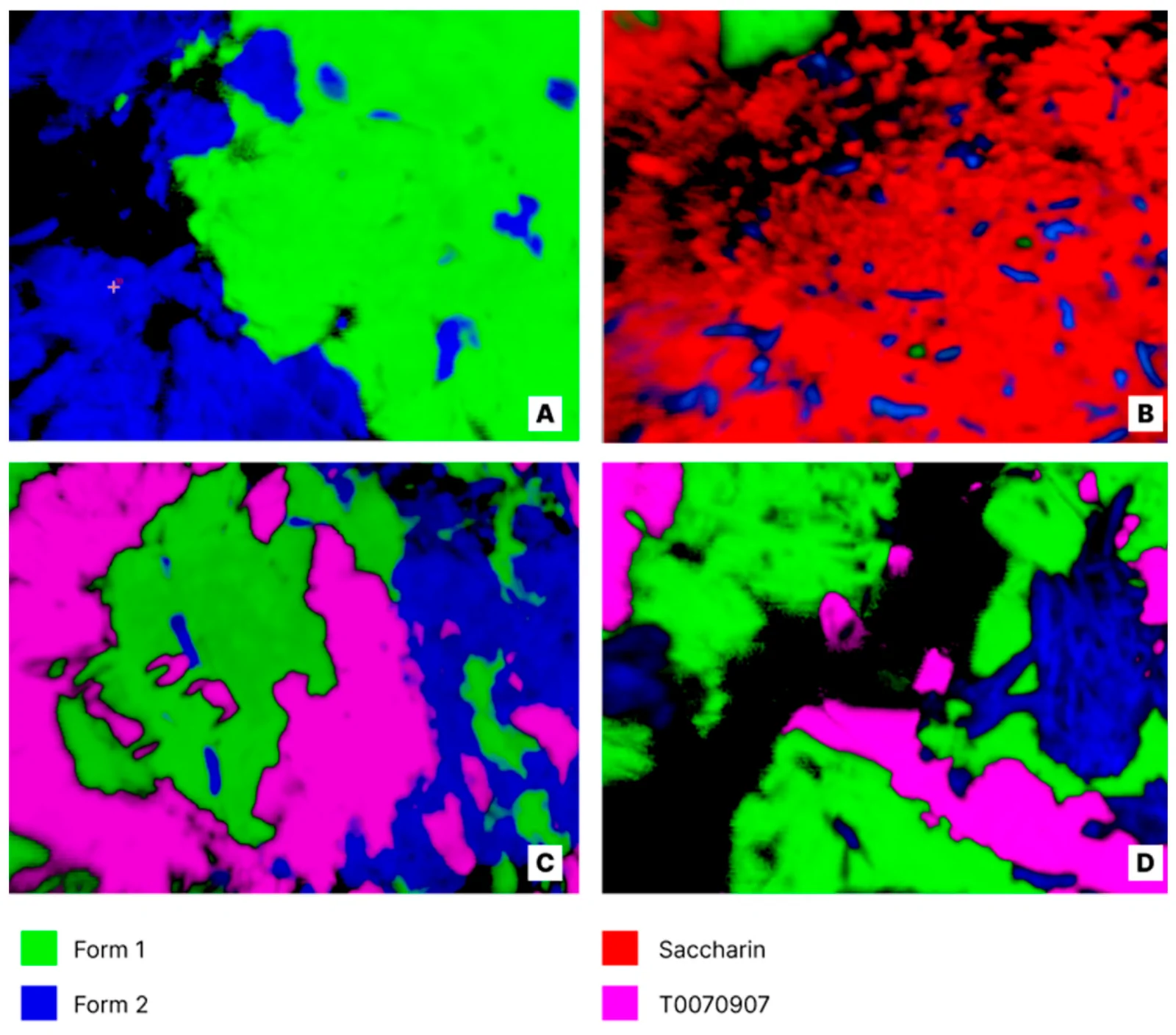
FTIR chemical images of freeze-dried T0070907–saccharin mixtures at different molar ratios. False-colour chemical maps of freeze-dried powders at saccharin/T0070907 molar ratios of 1:1 (**A**), 1.6:1 (**B**), 1:2 (**C**) and 1:3 (**D**), showing spatial distribution of both components and improved mixture homogeneity under saccharin excess (>1:1).

Chemical images of the mixtures (Figure 2) showed that for a saccharin-to-T0070907 molar ratio of 1.6:1, saccharin is in large excess, while T0070907 is fully solubilised and FTIR interaction signatures F1 and F2 appear. When T0070907 is in excess (1:2 or 1:3), saccharin is not detected (or only weakly detected) in the mapped area; FTIR interaction signatures F1 and F2 remain, but precipitation of pure T0070907 is likely. At a 1:1 ratio, FTIR interaction signatures F1 and F2 are observed, yet residual saccharin or T0070907 may persist, again risking precipitation. These observations guided the choice of a saccharin/T0070907 molar ratio > 1:1 for subsequent liquid formulation development. No stoichiometry is inferred from these images, which report spatial distribution only.

The solid-state organisation of the T0070907–saccharin systems was investigated by XRPD as a function of increasing saccharin content (Figure 2). T0070907 was used as the starting reference, followed by binary systems with increasing saccharin/T0070907 molar ratios of 1:3, 1:2, 1:1, 1.6:1, 2:1 and 3:1, and, finally, saccharin.

T0070907 exhibited a characteristic crystalline diffraction pattern, dominated by an intense reflection at 2θ ≈ 10.2°, together with additional reflections at approximately 15.1°, 20.5° and 25.9°. Upon addition of saccharin, substantial composition-dependent changes in the diffraction pattern were observed. At a saccharin/T0070907 molar ratio of 1:3, the main reflection at approximately 10.2° was retained, while additional reflections became apparent, notably at 2θ ≈ 13.3° and 26.8°. The latter became one of the dominant reflections of the binary system. Further changes were observed at the 1:2 ratio, with reflections at approximately 13.3°, 22.0° and 26.8° becoming prominent.

The most pronounced modification of the diffraction pattern occurred around the equimolar composition. The 1:1 system displayed a substantially different profile from both T0070907 and the T0070907-rich mixtures. In particular, the intense T0070907 reflection at 2θ ≈ 10.2° was no longer observed as such, whereas pronounced reflections appeared at approximately 9.6°, 11.7°, 14.4°, 26.4° and 26.8°. Several [FXL1.1]other reflections of lower intensity were also observed throughout the diffraction pattern. This marked redistribution of the diffraction features, involving both the disappearance of reflections associated with the starting T0070907 solid state and the appearance of additional reflections, supports a major change in crystalline organisation.

**Figure 3:**
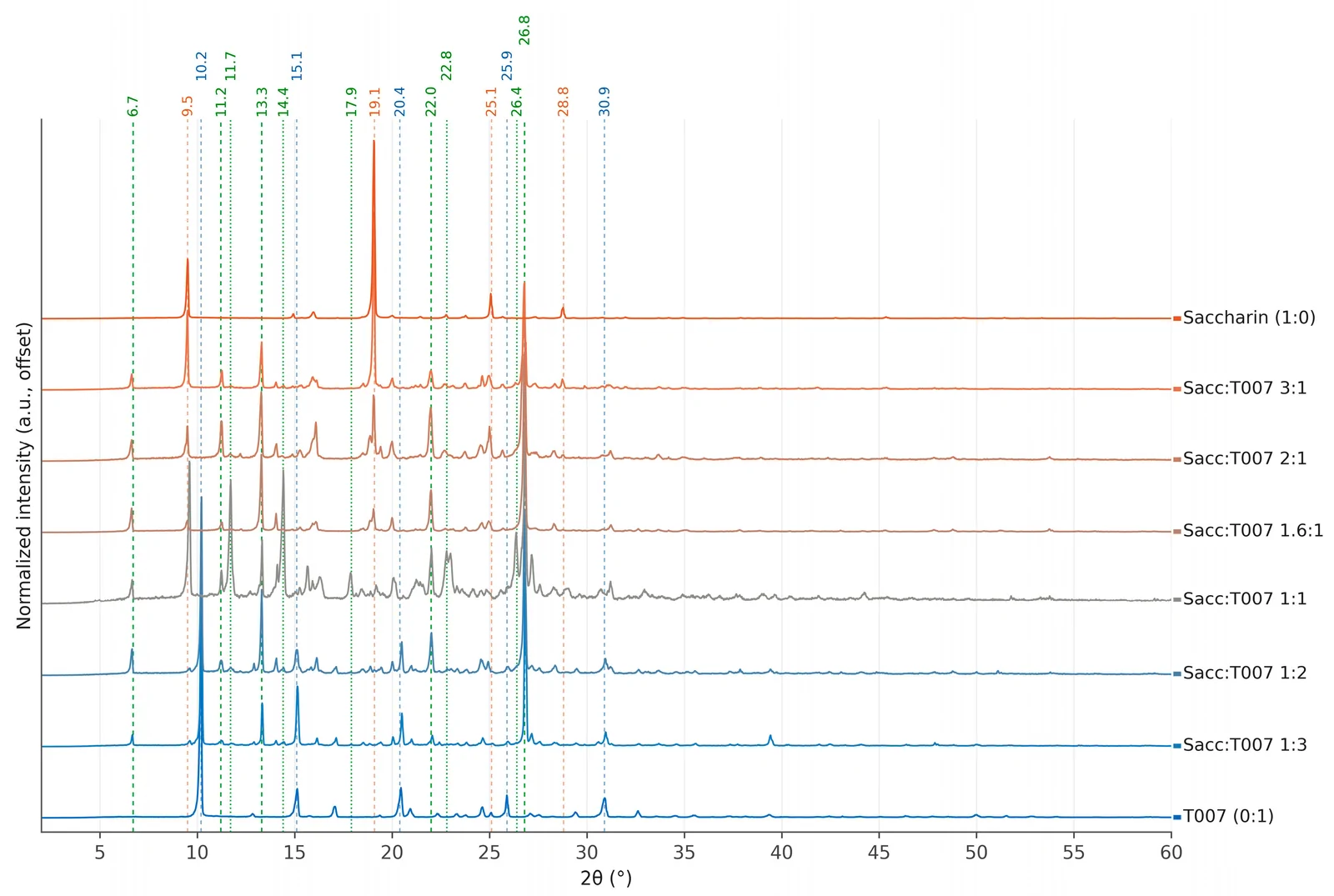
X-ray powder diffraction (XRPD) patterns of saccharin/T007 mixtures at different saccharin/T007 mass ratios (from 0:1 to 1:0). The samples were dissolved in water and then freeze-dried. Each pattern was min–max normalised and vertically offset by 0.4 for clarity. Dashed lines mark the characteristic reflections of saccharin (orange: 9.5, 19.1, 25.1 and 28.8° 2θ) and of T007 (blue: 10.2, 15.1, 20.4, 25.9 and 30.9° 2θ). Green dashed lines mark new reflections, absent from both pure components, that are seen in most mixtures (6.7, 11.2, 13.3, 22.0 and 26.8° 2θ). Green dotted lines mark the reflections found only in the 1:1 mixture (11.7, 14.4, 17.9, 22.8 and 26.4° 2θ).

Increasing the saccharin/T0070907 ratio to 1.6:1 resulted in a further modification of the diffraction pattern. The complex series of reflections observed for the 1:1 system was markedly reduced, while reflections at approximately 13.3°, 22.0° and 26.8° became prominent. The persistence of reflections around 13.3°, 22.0° and 26.8° over several intermediate compositions, together with the appearance and disappearance of other reflections depending on composition, indicates the presence of additional crystalline phase(s) in the binary mixtures, whose relative contribution strongly depends on the saccharin/T0070907 molar ratio (Figure 3).

At higher saccharin contents, the diffraction patterns progressively evolved towards that of saccharin. At ratios of 2:1 and 3:1, reflections around 2θ ≈ 9.5° and 19.1° became increasingly prominent. In particular, the reflection at approximately 19.1° became the dominant feature of the 3:1 pattern, consistent with the major reflection of saccharin at 2θ ≈ 19.1°. Nevertheless, the 2:1 and 3:1 patterns remained distinguishable from that of saccharin, notably through the persistence of reflections around 13.3°, 22.0° and 26.8°.

Overall, the XRPD series revealed a non-linear evolution of the crystalline organisation upon progressive addition of saccharin to T0070907, characterised by the appearance and disappearance of several diffraction reflections and marked changes in their relative intensities. The particularly substantial changes observed at intermediate compositions, together with diffraction features that cannot be accounted for solely by the patterns of the individual components, support the formation of additional crystalline phase(s) whose relative contribution varies with the saccharin/T0070907 molar ratio.

### 3.3. Saccharin-Based Oral Solution

#### 3.3.1. Composition

Based on the solubility and FTIR data, a saccharin-based oral solution was designed using PEG 400 and propylene glycol as co-solvents and polyvinylpyrrolidone (PVP) as a crystallisation inhibitor. The final formulation contained 1.8% (*w*/*w*) T0070907 (20 mg/mL), 1.8% saccharin, 3.0% PVP K10 and 1.6% PVP K30 in a 1:1 (*w*/*w*) PEG 400/propylene glycol vehicle (Table 4). The pH was approximately 5.0, the saccharin/T0070907 molar ratio was 1.6:1 (1:1 *w*/*w*), and the solution was administered at 0.5 mL/kg to deliver a 10 mg/kg dose. Osmolality is not reported because the vehicle is non-aqueous (PEG 400/propylene glycol 1:1) and has no continuous aqueous phase, which makes freezing-point depression osmometry inapplicable. Freshly prepared solutions were clear, free from visible particles and remained limpid upon storage in amber glass vials.

The contribution of saccharin can be assessed from the co-solvent data of Table 2. T0070907 dissolves at 15.66 mg/mL in neat PEG 400 and at 4.69 mg/mL in neat propylene glycol; for the 1:1 (*w*/*w*) mixture used here, log-additive behaviour predicts an apparent solubility of approximately 9–10 mg/mL, less than half the 20 mg/mL achieved in the final formulation. PVP K10 and K30, used at 3.0% and 1.6% as crystallisation inhibitors, are not expected to provide a co-solvent effect of sufficient magnitude to account for the observed increase in solubility. Consistent with a specific interaction rather than a medium effect, saccharin salts, in which the saccharinate anion can no longer donate a proton, caused immediate precipitation of T0070907 under otherwise identical conditions. Additional measurements of T0070907 solubility in the saccharin-free vehicle and across a range of saccharin concentrations would further refine this assessment, as discussed in Section 4.4.

The demonstrated 90-day room-temperature stability in amber glass vials is compatible with preparation and storage in a hospital pharmacy setting.

#### 3.3.2. Validation of the Stability-Indicating UHPLC and LC–MS Methods

The UHPLC method developed for routine assay and stability studies of T0070907 showed excellent linearity over the validated range (mean R^2^ = 0.9999) and acceptable repeatability and intermediate precision across three QC levels (25, 75 and 110 µg/mL). Trueness ranged from 98.9% to 102.0%, and the LLOQ was 10 µg/mL, set as the lowest concentration giving a signal-to-noise ratio of at least 10 and meeting the ICH Q2(R2) accuracy and precision criteria.

The LC–MS method used for quantification of T0070907 in biological matrices also fulfilled ICH Q2(R2) criteria. Linearity (R^2^ = 0.9998) was demonstrated over the 25–450 ng/mL range, with good repeatability and intermediate precision at each QC level. Trueness values were within 97.8–101.1%, and the LLOQ and LOD were 109.88 ng/mL and 36.26 ng/mL, respectively. This method was used for formulation assay and stability studies, as well as for exploratory analysis of biological samples; its sensitivity in plasma was not sufficient to draw conclusions regarding systemic exposure (Section 3.4.1).

**Figure 4:**
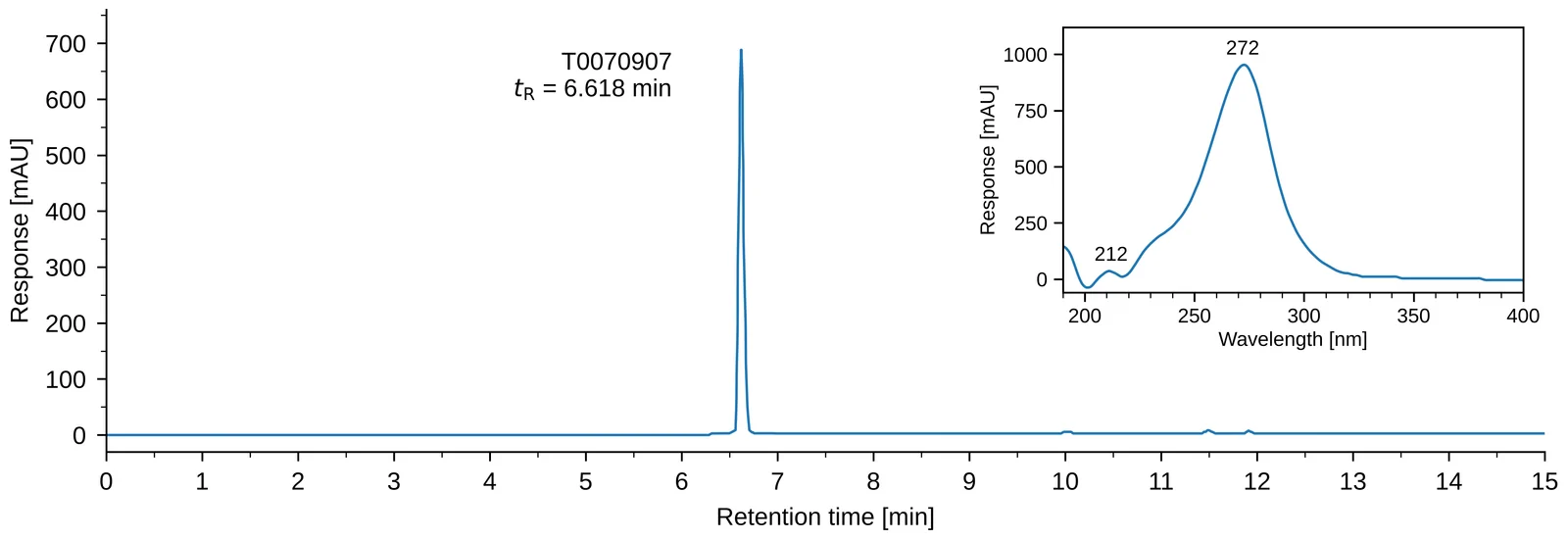
Representative UHPLC chromatogram and UV absorbance spectrum of T0070907 (0.1 mg/mL) in the saccharin-based oral solution. Chromatogram recorded at 254 nm showing the T0070907 peak and the absence of interference from formulation excipients, and UV spectrum of T0070907 in the mobile phase, recorded during UHPLC analysis, illustrating the main absorption maxima used for quantification.

**Figure 5:**
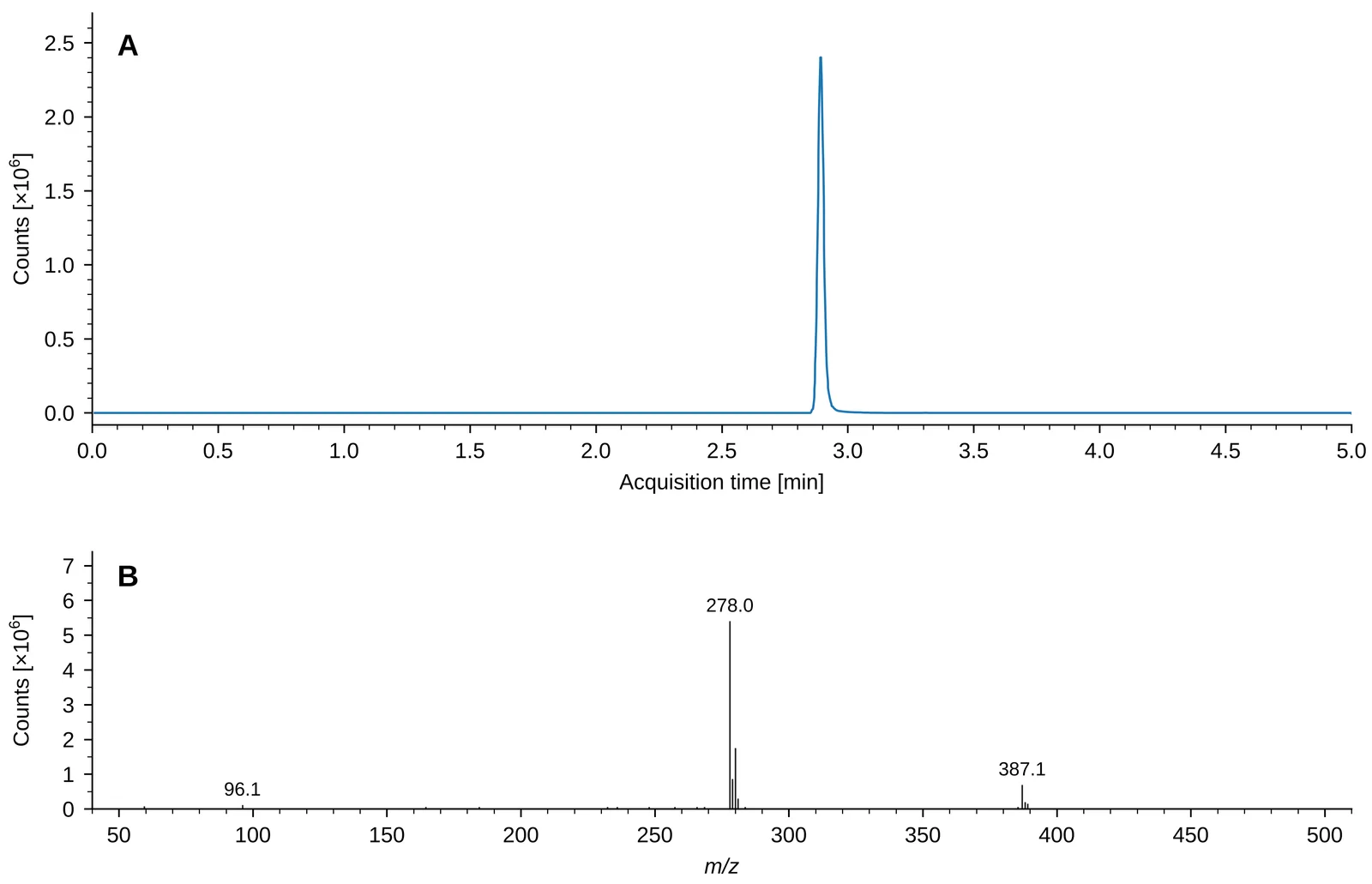
Representative LC–MS chromatogram of T0070907 at 5 µg/mL (**A**) and mass spectrum of T0070907 showing the ion at *m*/*z* 278 (**B**).

Among the excipients used in the formulation, only saccharin exhibited UV absorbance near the analytical wavelength selected for T0070907; however, it did not interfere with the T0070907 peak or affect method performance. Representative UV and MS chromatograms of T0070907, together with the corresponding UV and mass spectra, are shown in Figure 4 and Figure 5.

#### 3.3.3. Forced Degradation

**Figure 6:**
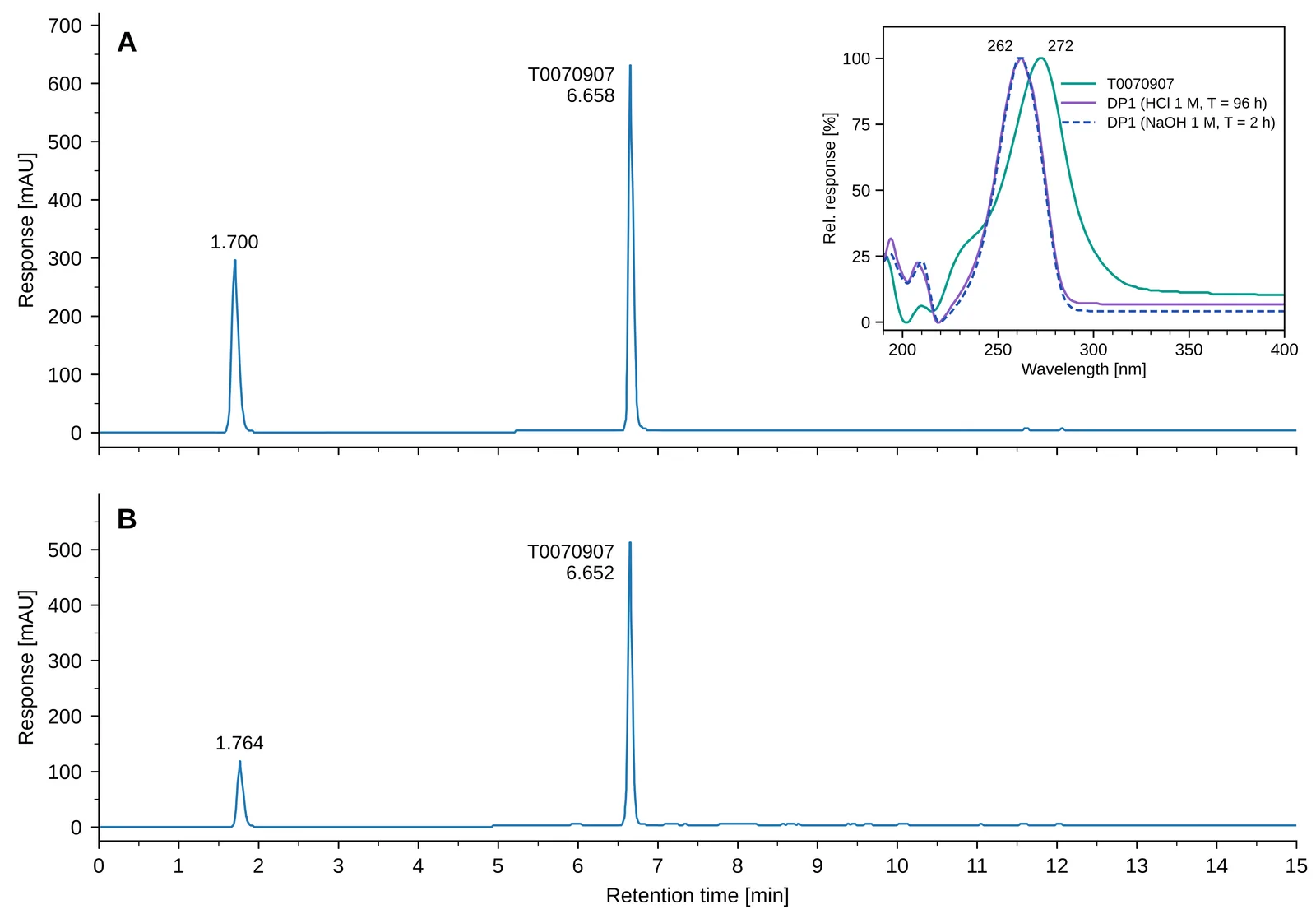
UHPLC chromatograms of T0070907 under forced degradation, showing the major degradation product DP1 (retention time ~1.7–1.8 min) alongside the residual T0070907 peak (~6.6 min): (**A**) 1 M HCl stress; (**B**) 1 M NaOH stress. Inset in panel A: overlaid UV absorbance spectra of T0070907 and DP1 generated under acidic and alkaline stress conditions, illustrating the spectral shift used to distinguish DP1 from the parent compound.

Forced degradation studies showed that T0070907 was stable in neutral solution but degraded under strong acidic and basic conditions. Exposure to 1 M HCl or 1 M NaOH at elevated temperature led to progressive loss of the parent peak and the appearance of one dominant degradation product (DP1) at a retention time of 1.76 min (Figure 6). The UV spectrum of DP1 differed markedly from that of T0070907, suggesting a structural modification of the aromatic system. Under oxidative conditions (3% H_2_O_2_), T0070907 proved markedly more stable, with no formation of DP1 or additional degradation products over the study period. Overall, the forced degradation profile demonstrated that the UHPLC method was stability-indicating, capable of separating T0070907 from its main degradation product under relevant stress conditions.

**Figure 7:**
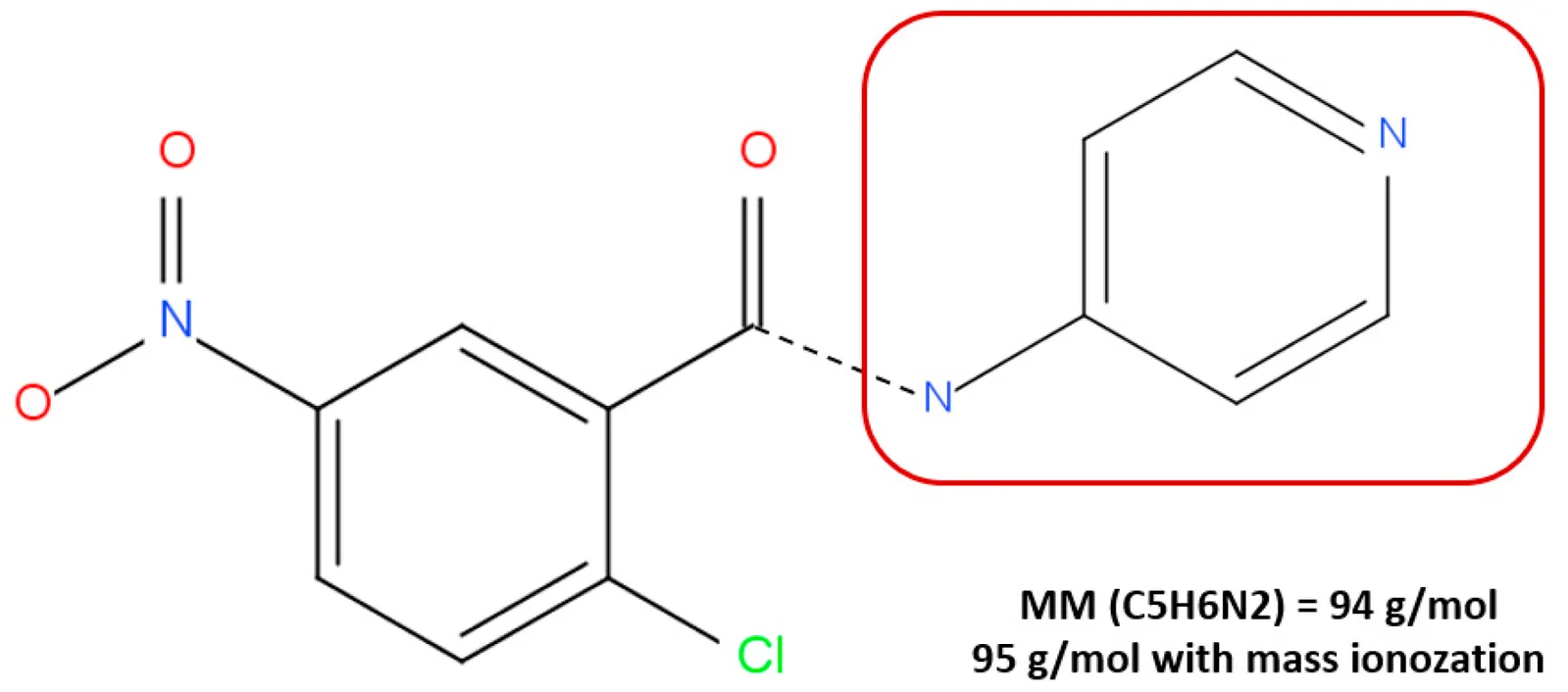
Identification of the major degradation product DP1 by LC–MS. Cleavage of the amide bond of T0070907 (dashed bond) results in the formation of pyridin-4-amine (C_5_H_6_N_2_, monoisotopic mass: 94 g/mol), consistent with the LC–MS ion observed for DP1 at *m*/*z* 95 [M + H]^+^ under acidic and alkaline stress conditions. The identity of DP1 was confirmed by comparison with an authentic 4-aminopyridine reference standard.

LC–MS analysis of the stressed samples identified a major degradation product with an [M + H]^+^ ion at *m*/*z* 95 (neutral molecular mass: 94 g/mol; elemental composition: C_5_H_6_N_2_). This molecular mass is consistent with cleavage of the amide bond linking the 2-chloro-5-nitrobenzoyl moiety to the pyridin-4-yl nitrogen of T0070907, resulting in the formation of pyridin-4-amine (Figure 7). DP1 was identified as pyridin-4-amine (4-aminopyridine, CAS: 504-24-5), by comparison with an authentic reference standard. This degradation pathway is consistent with the susceptibility of the amide bond to hydrolysis under the acidic and alkaline forced degradation conditions investigated.

#### 3.3.4. Long-Term Stability of the Oral Solution

The saccharin-based oral solution (20 mg/mL T0070907) remained clear and free from visible particulates throughout 90 days of storage at room temperature in amber glass containers. In a complementary compatibility study, a placebo formulation with an identical excipient composition was filled into hard capsules and stored under the same conditions, with no evidence of leakage or alteration of capsule integrity. The 90-day stability study of T0070907 was conducted in amber glass vials, while the capsule experiment was designed specifically to assess the physical compatibility of the vehicle with the capsule shell. Accordingly, these experiments provide complementary information on formulation stability and capsule compatibility, respectively.

Quantitative analysis showed that T0070907 content remained close to the initial value over the 90-day period (Table 5). The chromatographic profile remained simple, with DP1 detected at low levels and no additional degradation products emerging during storage. The pH of the solution remained stable (approximately 5.0). All time points met the predefined acceptance criteria (content within 90–110% of the initial value, no visible particles or colour change, pH within ±0.5 units). These results demonstrate that the saccharin-based formulation provides a chemically and physically stable oral solution of T0070907 over at least three months.

### 3.4. In Vivo Biopharmaceutics

#### 3.4.1. Rodent Systemic and Tissue Exposure (LC–MS/MS)

In the in-house study, mice received repeated oral dosing of the saccharin-based formulation (10 mg/kg, once daily), and plasma and bone samples were collected 1 h after the final dose. Parent T0070907 was not detected above the lower limit of quantification of the in-house assay in plasma, while the internal standard confirmed assay performance. That assay has an LLOQ of 109.88 ng/mL in plasma, about fifty times higher than the LC–MS/MS method used in the Eurofins studies; it can therefore exclude high plasma concentrations but cannot demonstrate low exposure, and this dataset is not used here to support any conclusion on systemic exposure. A T0070907-related signal was detected in bone from treated animals and was absent in control animals. Because the extraction yield from bone powder was approximately 7%, estimated from three spiked control samples with a single-point correction and without matrix-matched calibration, these bone measurements are reported as a qualitative observation only; individual values per animal are given in the Supplementary Materials and are explicitly semi-quantitative.

In the first CRO study, parent T0070907 was below the limit of quantification in plasma at all time points following oral dosing of the saccharin-based formulation (5 or 10 mg/kg) and was detectable only at the earliest time point after IV dosing with the same formulation (1 mg/kg). No parent compound was detected in femur at 24 h.

The second CRO study was designed to determine whether quantifiable systemic exposure to T0070907 could be achieved when administered in a vehicle selected to maximise drug solubilisation: T0070907 was therefore administered in DMSO rather than in the saccharin-based formulation, with the aim of maximising the amount of dissolved drug available for absorption in the gastrointestinal tract. Following oral dosing at 100 mg/kg in DMSO, parent T0070907 remained undetectable in plasma and most tissues. Sporadic low-level detections were observed in liver (4 ng/g) and thigh muscle (26 ng/g), while kidney, brain, and remaining tissues were below the limit of quantification.

Across the two CRO studies, parent T0070907 remained below the limit of quantification in plasma at all sampling times following oral administration at 5, 10 and 100 mg/kg, so that no pharmacokinetic parameters (C_max_, T_max_, AUC, terminal half-life, apparent clearance or oral bioavailability) could be determined. Measurable plasma concentrations of parent T0070907 were obtained only at the earliest sampling times after intravenous administration. Because the high oral dose delivered in DMSO also failed to produce quantifiable parent exposure, the lack of exposure cannot be attributed to the saccharin formulation or to precipitation from it.

#### 3.4.2. Hepatic Metabolism in Human Liver Microsomes

The metabolic stability of T0070907 was evaluated in human liver microsomes. Under the conditions used (PBS pH 7.4, no co-solvent), the limited aqueous solubility of T0070907 resulted in partial precipitation during the experiment. A time-dependent decrease in T0070907 peak area was observed in incubations containing microsomes and NADPH; however, a decrease was also observed to some extent in control incubations without microsomes, indicating that the loss of parent compound could not be attributed exclusively to microsomal metabolism. Chemical instability and precipitation may also have contributed to the observed decrease. Moreover, no major, well-defined metabolite peaks could be reliably identified under these conditions. Consequently, the microsomal experiments did not allow a reliable assessment of intrinsic metabolic stability for highly insoluble and chemically sensitive compounds such as T0070907 under conditions requiring low co-solvent levels and neutral pH.

### 3.5. Genotoxicity Assessment

#### 3.5.1. In Silico Predictions

Given the nitroaromatic structure of T0070907 and the formation of a major degradation product (DP1), the potential mutagenicity risk was assessed in accordance with ICH M7 [17]. In silico tools (Toxtree and T.E.S.T.) flagged T0070907 as potentially mutagenic, prompting further experimental evaluation using the Ames test. DP1, identified by LC–MS as pyridin-4-amine (Section 3.3.3) and confirmed by comparison with an authentic reference standard, was also considered: primary aromatic amines are a well-recognised structural alert for bacterial mutagenicity under the ICH M7 framework, as they can undergo metabolic N-hydroxylation to form reactive nitrenium-ion intermediates capable of interacting with DNA. DP1 was therefore considered to carry a structural alert for mutagenicity that is distinct from the nitroaromatic alert associated with the parent compound.

#### 3.5.2. Ames Bacterial Reverse Mutation Test

In the Ames test, T0070907 was evaluated in five *Salmonella typhimurium* strains (TA1535, TA1537, TA98, TA100 and TA102) both in the presence and absence of metabolic activation (S9 mix). Cytotoxicity and limited solubility restricted the highest testable concentrations but allowed multiple concentration levels to be evaluated below those associated with overt cytotoxicity.

In both independent experiments, the vehicle and positive controls produced responses within the corresponding historical control ranges. Background lawn growth and sterility controls were satisfactory under all test conditions. T0070907 induced a clear, concentration-related increase in revertant counts in several tester strains, both in the presence and absence of S9 mix, while other strains remained within or close to the corresponding negative control range. Based on these findings, and according to the predefined decision criteria, T0070907 was considered mutagenic under the conditions of the Ames test (Table 6). Importantly, the test item was the unstressed commercial batch (purity > 99%), in which DP1 was not present at a relevant level. The observed mutagenic response can therefore be primarily associated with the parent compound rather than with DP1 generated during degradation. Detailed revertant counts for each strain, metabolic activation condition, concentration and replicate are provided in Supplementary Data Table S1.

## 4. Discussion

This work was designed as an integrated “developability snapshot” of T0070907, a nitrobenzamide PPARγ antagonist with promising antitumour activity but essentially no prior pharmaceutical characterisation. Physicochemical profiling, formulation screening, saccharin-based supramolecular design, biopharmaceutical evaluation, and early genotoxicity testing were combined to assess whether T0070907 could realistically be considered a drug candidate or should remain primarily a pharmacological tool compound.

### 4.1. Saccharin as a Supramolecular Solubiliser and Formulation Enabler

The intrinsic properties of T0070907—very low aqueous solubility, high crystallinity and limited solubility in pharmaceutically acceptable solvents—are typical of late-discovery tool molecules and are unfavourable for oral drug development. Conventional solubilisation strategies, including co-solvents, surfactant- and lipid-based systems or cyclodextrin inclusion, failed to achieve the target concentration required for meaningful dosing in preclinical models. Table 2 and Table 3 therefore provide a direct comparison of saccharin with three established solubilisation strategies, namely co-solvency, surfactant- and lipid-based systems and cyclodextrin complexation, all evaluated under comparable experimental conditions. Even the best-performing solubilising excipients (PEG 400, PEG 600, Transcutol^®^, Tween^®^ 20 and Tween^®^ 60) provided solubility values around 15–16 mg/mL, below the 20 mg/mL needed for a practical high-dose oral solution and raised concerns about viscosity and patient acceptability at the required excipient levels.

In contrast, saccharin markedly improved the apparent solubility of T0070907 in PEG/propylene glycol vehicles and allowed the development of a 20 mg/mL oral solution. FTIR imaging of freeze-dried T0070907–saccharin mixtures revealed clear spectral changes and two distinct FTIR interaction signatures (F1 and F2) consistent with drug–excipient interactions and distinct from either pure component. The shifts and intensity changes in bands associated with the sulfonamide, aromatic and carbonyl regions are consistent with hydrogen bonding and other non-covalent interactions between T0070907 and saccharin, supporting the formation of supramolecular complexes rather than a simple physical mixture. Infrared chemical mapping further showed that an excess of saccharin (molar ratio > 1:1) is required to avoid residual domains of pure T0070907 and minimise the risk of precipitation, which directly informed the final formulation design.

Saccharin is an attractive excipient in this context because it is pharmacopeial, widely used in oral products and can act as a strong hydrogen-bond acceptor while remaining sufficiently soluble to dissolve in PEG and propylene glycol. It is also a strong acid (pKa 1.6) and can act as a directional hydrogen-bond donor towards the weakly basic pyridine nitrogen of T0070907, a combination of properties not shared by the other solubilisation approaches evaluated here. The immediate precipitation observed with saccharin salts, in which the saccharinate anion can no longer donate a proton, further supports the importance of neutral saccharin in the observed interaction. The saccharin/T0070907 molar ratio of 1.6:1 (mass ratio 1:1) used in the final formulation therefore favours supramolecular association while maintaining saccharin exposure within recommended daily intake limits [23]. The XRPD patterns show that adding saccharin markedly changes the solid-state organisation of T0070907. They cannot be described as a simple superposition of the two components. As the saccharin content increased, new reflections appeared, others weakened or disappeared, and relative intensities changed. These changes were already visible in the T0070907-rich 1:3 and 1:2 systems, so even small amounts of saccharin affect T0070907 crystallisation. The 1:1 composition gave a distinct pattern, which again differed from that of the 1:6:1 system. The 1:1 system is therefore not a simple intermediate between the two ends of the series. At 2:1 and 3:1, the saccharin reflections became dominant, but additional reflections persisted. Overall, these results support the formation of one or more additional crystalline phases whose contribution depends on composition. This is consistent with the FTIR imaging data, which point to specific interactions between the two compounds. Structural characterisation will nevertheless be needed to establish whether a defined cocrystal forms, and to determine its stoichiometry. Overall, these findings support the concept of saccharin as a functional supramolecular solubiliser and suggest its potential utility in the formulation of other poorly soluble nitrobenzamides and related heteroaromatics.

These findings complement previous reports of saccharin and saccharinate salts forming co-crystals or salts with basic APIs to enhance solubility and dissolution, but extend this concept to a poorly water-soluble nitrobenzamide formulated as a PEG/PG oral solution rather than as a classical solid oral dosage form [10,24].

The resulting oral solution showed good physical and chemical stability over 90 days at room temperature when stored in amber glass vials. In a complementary compatibility experiment, the corresponding placebo vehicle showed no leakage or alteration of hard-capsule integrity under the same storage conditions, supporting the feasibility of capsule filling to mitigate viscosity and taste issues. Forced degradation studies revealed one major low-molecular-weight degradation product (DP1) under both acidic and alkaline conditions. LC–MS analysis identified DP1 as pyridin-4-amine ([M + H]^+^ at *m*/*z* 95 Da; monoisotopic neutral mass: 94 Da), arising from hydrolytic cleavage of the T0070907 amide bond, with its identity confirmed by comparison with an authentic reference standard. The identification of a major, structurally rationalised degradation pathway facilitates future analytical quality control and provides a defined impurity for consideration within an ICH M7-based risk assessment.

### 4.2. Biopharmaceutics and Disposition: A Complex Picture

The in vivo datasets underscore the challenges of translating T0070907 beyond its historical role as a tool compound. Exposure conclusions in this section rest exclusively on the Eurofins rat studies (LLOQ 2 ng/mL in plasma, 2 ng/g in tissue homogenates); the in-house mouse dataset is not sensitive enough to contribute to them. Using a sensitive rat LC–MS/MS method, parent T0070907 was generally not quantifiable following oral dosing across two studies, and no quantifiable levels were detected in femur collected at 24 h. In the early distribution snapshot study (1 h), most analysed tissues, including the kidney, brain, liver and thigh muscle, showed no quantifiable parent compound in nearly all animals, with only sporadic low-level detections in individual animals (e.g., liver 4 ng/g; thigh muscle 26 ng/g). Collectively, these findings are consistent with very low systemic exposure to the parent compound after oral dosing under the tested conditions, and suggest that any parent exposure—if present—is likely to be limited and/or transient and not reliably captured by parent-only measurements.

Importantly, the absence of quantifiable parent concentrations does not discriminate among several non-mutually exclusive mechanisms, including limited absorption, precipitation in the gastrointestinal tract despite formulation-enabled dosing, rapid first-pass loss, and/or conversion to metabolites not captured by a parent-focused assay. The intermittent low-level detections in organs at 1 h suggest that brief or heterogeneous exposure may occur in some animals; however, the overall pattern remains dominated by non-quantifiable measurements and does not support sustained or reproducible parent exposure in plasma or tissues at the investigated doses and time points.

In vitro metabolism studies in human liver microsomes were similarly constrained by the same physicochemical limitations that motivated the formulation work. Maintaining low organic solvent levels and near-neutral pH for microsomal activity limited apparent solubility and led to precipitation during incubation. Under these conditions, declines in the parent signal were observed, but could not be confidently attributed to enzymatic turnover versus non-specific loss (precipitation and/or chemical instability), and no major metabolites could be reliably assigned. This illustrates a general challenge with poorly soluble and chemically labile compounds: standard microsomal stability assays may be difficult to interpret unless solubility-enabling conditions and orthogonal controls are implemented.

From a development perspective, these results support two practical conclusions. First, the saccharin-based formulation enables standardised oral dosing of an otherwise intractable compound, but does not, by itself, guarantee quantifiable systemic exposure of the parent compound; the vehicle-based benchmark at 100 mg/kg in DMSO shows that this is not a limitation of the formulation. Second, understanding T0070907 disposition would require a broader analytical strategy (e.g., metabolite identification, lower-level quantification where feasible, and/or radiolabelled mass-balance approaches) if exposure-driven conclusions are needed.

Within the scope of the present work, the most robust inference is that parent T0070907 shows no quantifiable systemic or tissue exposure after oral dosing in rats at the doses and sampling times investigated, reinforcing its positioning primarily as a pharmacological tool and developability case study rather than a straightforward development candidate.

### 4.3. Early Genotoxicity as a Key Developability Filter

The nitroaromatic motif of T0070907 and the presence of a dominant degradation product naturally raised concerns under ICH M7. In silico assessment using two complementary tools (a rule-based expert system and a QSAR approach) flagged the parent compound as potentially mutagenic, which motivated an experimental Ames test. LC–MS fragmentation data identified DP1 as pyridin-4-amine, released by hydrolytic cleavage of the T0070907 amide bond; as a primary aromatic amine, this fragment carries its own recognised ICH M7 structural alert for bacterial mutagenicity, independent of the nitroaromatic alert borne by the parent compound. That alert is an in silico prediction under ICH M7 and was not tested experimentally, since isolated DP1 was not submitted to the Ames assay. In a GLP-compliant bacterial reverse mutation assay conducted according to OECD 471, T0070907 induced clear, concentration-related increases in revertants in TA98 and TA100, both with and without metabolic activation, while the other strains remained negative. The pattern of response, positive in both a frameshift and a base-substitution strain, is consistent with aromatic nitro compounds that can undergo reductive activation in bacteria.

The combination of concordant in silico alerts and a positive Ames test strongly supports classifying T0070907 as mutagenic in bacteria. For many therapeutic areas, this would effectively stop development of the molecule itself. In oncology, a mutagenic profile is not necessarily disqualifying, provided that the expected benefit is substantial, that exposure is limited in duration and that appropriate non-clinical follow-up confirms an acceptable overall genotoxic risk; any such argument would have to rest on demonstrated efficacy and exposure, neither of which is established here. Importantly, the mutagenicity of both the API and its main degradation product would also necessitate tight control of impurity levels and robust stability throughout the product’s shelf life.

The pattern of response is informative about the mechanism. Positive results were obtained in TA98, which detects frameshift events, and in TA100, which detects base-pair substitutions, both in the presence and in the absence of S9, while TA1535, TA1537 and TA102 remained negative. Independence from exogenous metabolic activation points to reductive bioactivation by endogenous bacterial nitroreductases rather than to cytochrome P450-mediated activation, following the sequence classically described for nitroaromatic compounds: reduction in the nitro group to the nitroso and N-hydroxylamine intermediates, O-esterification, and formation of an electrophilic nitrenium ion able to form DNA adducts. The negative response in TA102, which is sensitive to oxidative and cross-linking agents, is consistent with a mechanism that is not primarily oxidative [25,26].

From a development standpoint, our results suggest two realistic paths for T0070907. It could be used primarily as a pharmacological tool to validate the therapeutic relevance of PPARγ antagonism in osteosarcoma and other tumours, while clinical development efforts shift toward safer chemotypes. The second would be to restrict T0070907 to preclinical research and teaching, where it could serve as a didactic example of early developability and genotoxicity assessment. Both are consistent with the data generated here; advancing the molecule itself as a therapeutic candidate is not.

In this context, the present work serves less to disqualify T0070907 outright than to clarify its risk profile and development trajectory. Our results suggest that T0070907 should be considered a mutagenic compound requiring a carefully justified risk–benefit balance, rather than a default “safe” candidate. In parallel, T0070907 remains an extremely valuable pharmacological tool to interrogate PPARγ signalling and to validate the therapeutic relevance of its antagonism in osteosarcoma and other tumours, while safer chemical series are explored.

### 4.4. Implications and Limitations

This study has several limitations. First, the solubility of T0070907 in the saccharin-free vehicle was not measured and no saccharin concentration series was generated; the contribution of saccharin is therefore supported by the bounding estimate given in Section 3.3.1, by the FTIR data and by the behaviour of saccharin salts, but not by a direct control experiment. Second, the in vivo work does not provide a pharmacokinetic characterisation: parent concentrations were below the limit of quantification at all sampling times after oral dosing, no parameter could be derived, and the measurements remain parent-focused, so they do not resolve the contribution of metabolites or transient exposure outside the sampled windows. Third, the bone data are semi-quantitative, and matrix-matched calibration in bone powder would be required before any quantitative conclusion could be drawn from this matrix. Fourth, the two FTIR interaction signatures were reproducible but coexisted within the same samples, and phase assignment, that is, the distinction between a salt, a cocrystal and an amorphous state, would require orthogonal solid-state analyses such as XRPD together with thermal methods or solid-state NMR. Fifth, the viscosity of the formulation was not measured. Sixth, the stability study was designed against the framework applicable to hospital preparations, and a full ICH Q1A(R2) programme with controlled temperature and humidity would be required for any further development. Finally, mutagenicity was used as an early filter and isolated DP1 was not tested; broader toxicology, including off-target pharmacology, safety pharmacology and organ toxicity, was not investigated.

In addition, efficacy testing in osteosarcoma models was outside the scope of this article and will need to be integrated with the present formulation, exposure and genotoxicity findings to evaluate the broader translational relevance of PPARγ antagonism in this setting.

Despite these limitations, the present work delivers practical messages. It demonstrates that saccharin can function as a supramolecular excipient to enable a robust, high-strength (20 mg/mL) oral formulation of a highly insoluble nitrobenzamide, supported by a stability-indicating control strategy and stress testing. More broadly, it illustrates how early integration of formulation development, sensitive exposure assessment and ICH M7-aligned genotoxicity triage can clarify both opportunities (standardised oral dosing for preclinical studies) and risks (mutagenicity and very low systemic exposure of the parent compound) when translating a widely used tool compound towards a development context. Accordingly, T0070907 is positioned here primarily as a pharmacological tool and as a didactic case study in early developability and genotoxicity risk assessment.

## 5. Conclusions

This work demonstrates that saccharin can act as a supramolecular excipient to enable a robust, high-strength oral liquid formulation of poorly soluble nitrobenzamide drugs such as T0070907. FTIR chemical imaging revealed two reproducible drug–excipient interaction signatures that guided formulation design, and a 20 mg/mL oral solution was developed and controlled using a validated stability-indicating UHPLC/LC–MS method. The formulation remained stable for at least 90 days under the tested storage conditions, with a single dominant stress-related degradation product (DP1) monitored as an impurity marker; DP1 was identified by LC–MS as pyridin-4-amine, released by hydrolytic cleavage of the T0070907 amide bond and confirmed against an authentic reference standard.

Despite formulation-enabled dosing, sensitive quantification in rats showed that parent T0070907 was below the limit of quantification in plasma at all sampling times after oral dosing, including at 100 mg/kg in a solubilising vehicle, so that no pharmacokinetic parameter could be derived. In parallel, an ICH M7-aligned triage supported by a GLP Ames test demonstrated that T0070907 is mutagenic in bacteria.

Taken together, these findings position saccharin-based supramolecular formulations as a practical approach to enable controlled dosing and developability assessment of poorly soluble nitrobenzamides, while supporting the use of T0070907 primarily as a pharmacological tool and as a case study illustrating formulation-enabled early developability and genotoxicity risk assessment.

## Figures and Tables

**Table 1:** Physicochemical properties of T0070907. Molecular formula, molecular weight, pKa values, log D_7.4_, melting point, degradation temperature and equilibrium solubility in selected solvents (water, methanol, DMSO and DMF).

Parameter	Value	Method/Comment
Chemical formula	C_12_H_8_ClN_3_O_3_	—
Molecular weight	277.6 g/mol	—
pKa_1_ (pyridine N, conjugate acid)	5.4	pH-metric titration in MeOH/water
pKa_2_ (amide N–H)	14.2	pH-metric titration in MeOH/water
log D_7.4_	1.76	in 1-octanol/HEPES buffer
Melting point	260 °C	HyperDSC
Onset of thermal degradation	185 °C	TGA
Water solubility (22–25 °C)	<0.1 mg/mL (practically insoluble)	UHPLC quantification
Methanol solubility (22–25 °C)	5 mg/mL	UHPLC quantification
DMSO solubility (22–25 °C)	60–90 mg/mL	UHPLC quantification
DMF solubility (22–25 °C)	100–140 mg/mL	UHPLC quantification

**Table 2:** Solubility of T0070907 in pharmaceutically acceptable solubilising vehicles. Equilibrium solubility (mean ± CV, *n* = 3) of T0070907 in Labrafil^®^ M 1944 CS, acidified water (pH 2), propylene glycol, Kolliphor^®^ EL, Labrasol^®^ ALF, Tween^®^ 20, Transcutol^®^ HP, Tween^®^ 60, PEG 400 and PEG 600 at room temperature.

Solubilising Vehicle	T0070907 Solubility (mg/mL) (*n* = 3)	CV (%)
Labrafil^®^ M 1944 CS	1.07	0.00
Water (pH 2)	2.74	0.06
Propylene glycol	4.69	0.09
Kolliphor^®^ EL	8.13	0.01
Labrasol^®^ ALF	11.41	0.06
Tween^®^ 20	12.62	0.78
Transcutol^®^ HP	13.56	1.24
Tween^®^ 60	15.27	0.13
PEG 400	15.66	0.04
PEG 600	15.80	0.08

**Table 3:** Solubility of T0070907 after cyclodextrin inclusion. Apparent solubility of T0070907 in aqueous solutions of SBE-β-CD, HP-β-CD and HP-γ-CD at a 1:5 T0070907:cyclodextrin molar ratio.

Cyclodextrin (CD)	T0070907:CD Molar Ratio	T0070907 Solubility (mg/mL)
SBE-β-CD (sulfobutylether-β-CD)	1:5	1.47
HP-β-CD (hydroxypropyl-β-CD)	1:5	1.28
HP-γ-CD (hydroxypropyl-γ-CD)	1:5	1.13

**Table 4:** Composition of the saccharin-based oral solution of T0070907. Qualitative and quantitative composition of the 20 mg/mL oral solution, expressed as grams per 100 mL and mass ratios (% *m*/*m*), with the functional role of each excipient (co-solvent, T0070907 solubilisation, crystallisation inhibition).

Excipient	For 100 mL of Solution (g)	Ratio (% *m*/*m*)	Role
Propylene glycol	50.00	45.9%	Saccharin solubiliser
PEG 400	50.00	45.9%	Saccharin solubiliser
Polyvinylpyrrolidone K10	3.30	3.0%	Crystallisation inhibitor
Polyvinylpyrrolidone K30	1.70	1.6%	Crystallisation inhibitor
Saccharin	2.00	1.8%	T0070907 solubiliser
T0070907	2.00	1.8%	API

**Table 5:** Long-term stability of T0070907 in the saccharin-based oral solution. Time course of T0070907 content (% of initial) in the oral solution stored at room temperature in amber glass vials over 90 days (*n* = 3).

Day	D0	D7	D14	D21	D28	D35	D42	D60	D90
T0070907 (%)	100	102	102	104	102	102	96	102	97
CV (%)	2.1	3.1	4.1	2.5	2.7	2.1	2.1	2.8	2.7

**Table 6:** Summary of Ames test outcomes for T0070907 (Pos = mutagenic, Neg = non-mutagenic according to predefined criteria).

Strain	−S9	+S9
TA1535	Neg	Neg
TA1537	Neg	Neg
TA98	Pos	Pos
TA100	Pos	Pos
TA102	Neg	Neg

## Data Availability

The data presented in this study are available on request from the corresponding author.

## Supplementary Materials

The following supporting information can be downloaded at: https://www.mdpi.com/article/10.3390/pharmaceutics18101258/s1, Figure S1: Effect of T0070907 on the thermotropic behaviour of DMPC (left) and DPPC (right) bilayers assessed by differential scanning calorimetry; Figure S2: AMES results; Table S1: AMES raw datas.
