# Supplementary material for: Saccharin-Enabled Oral Formulation, Biopharmaceutics and Early Genotoxicity Assessment of the PPARγ Antagonist T0070907
Source: Pharmaceutics. 2026 Oct 5;18(10):1258. doi: 10.3390/pharmaceutics18101258 (PMC13708121; doi:10.3390/pharmaceutics18101258)
Supplement: Supplementary file 1 [file pharmaceutics-18-01258-s001.zip › pharmaceutics-4527210-supplementary.pdf]

Supplementary Materials: Animals and Husbandry. Male Sprague-Dawley rats (BioLASCO Taiwan, under Charles River licence; 180 ± 20 g or 220 ± 20 g for tolerability, 180–250 g for PK; conventional, healthy, procedure-naïve) were acclimatised for 3 days and group-housed (three per cage, 45 × 23 × 21 cm or 47 × 25 × 21 cm) at 20–24 °C, 30–70 % relative humidity, 12 h/12 h light/dark, with ad libitum standard diet (MFG, Oriental Yeast) and autoclaved water.

Three exploratory in vivo experiments were conducted: a repeated-dose exposure study in mice (in-house) and, at Pharmacology Discovery Services Taiwan Ltd. (Eurofins Discovery partner lab, AAALAC-accredited), a rat acute tolerability (MTD) study and two rat pharmacokinetic studies. The mouse study was approved under authorisation APAFIS#27183-2020091414229028 (EU Directive 2010/63/EU); the rat studies were approved by the local IACUC (studies ODS112-01061300 and ODS112-01077544) and performed in accordance with the *Guide for the Care and Use of Laboratory Animals*, 8th ed.

Animals received T0070907 at 10 mg/kg once daily by oral gavage in the saccharin-based formulation for 20 to 35 consecutive days (n = 15). Control animals received an equivalent volume of vehicle alone. On the final day of dosing, animals were euthanised 1 h after administration. Blood samples were collected into heparinised tubes and centrifuged to obtain plasma, which was stored at −20 °C until analysis. Hind limb bones were harvested, cleared of soft tissues, frozen at −20 °C, and processed as described below.

To confirm these findings, complementary in vivo studies were performed by Eurofins to evaluate systemic and tissue exposure to the parent compound T0070907, using an LC–MS/MS method adapted from that developed by our unit (LLOQ: 2 ng/mL in plasma; 2 ng/g in tissue homogenates). Two rat studies were performed. In the first study, male Sprague-Dawley rats received T0070907 by oral gavage (5 or 10 mg/kg) or intravenous injection (1 mg/kg) to characterise plasma pharmacokinetics. Serial plasma samples were collected up to 24 h post-dose (oral: 0.167–24 h; IV: 0.05–24 h). Femurs were collected at study termination, homogenised, and analysed by LC–MS/MS.

In the second study, early plasma exposure was assessed following oral dosing at 100 mg/kg or intravenous dosing at 2 mg/kg of T0070907 dissolved in DMSO, with plasma samples collected from 0.05 to 1 h post-dose. At 1 h post-dose, animals were euthanised and tissues (liver, kidney, brain, and thigh muscle) were collected, rinsed with saline, weighed, and homogenised in PBS (pH 7.4) prior to LC–MS/MS analysis. Blood samples were collected into lithium-heparin tubes, maintained on ice, centrifuged within 1 h, and plasma and tissue homogenates were stored frozen (≤ −70 °C) until analysis. The detailed protocol is presented in the supplementary protocol.

Supplementary Materials: Animals

Acute tolerability (rats, n = 6 per group). Single doses of T0070907 were given IV at 10 mg/kg (2 mg/mL, 5 mL/kg) or PO at 100 mg/kg (10 mg/mL, 10 mL/kg); because 10 mg/kg IV was not tolerated, a second round tested 5 mg/kg IV (1 mg/mL in 0.9 % NaCl, 5 mL/kg) in back-up animals. Animals were unfasted. The primary outcome was tolerability (absence of mortality and of body-weight loss over 4 days); secondary outcomes were Irwin-type behavioural, neurological and autonomic signs scored from 0 to ±3, observed over the first 15 min and at 1 h post-dose (IV) or over the first 1 h and at 2 h post-dose (PO) on Day 1. Body weight and mortality were recorded daily to Day 4. Pre-defined humane endpoint: animals in distress or moribund were euthanised at the earliest such time point.

PK study 1—plasma and bone (rats, n = 6 per group). The saccharin-based 20 mg/mL solution was diluted in water for injection to 0.5 and 1 mg/mL (PO, 10 mL/kg, 5 and 10 mg/kg) and 0.2 mg/mL (IV, 5 mL/kg, 1 mg/kg); all solutions were clear, colourless, freshly prepared and light-protected at 20–25 °C. Several days beforehand, a jugular catheter was implanted under pentobarbital anaesthesia (50 mg/kg IP), with meloxicam (1 mg/kg SC) as post-operative analgesia, to permit serial sampling and reduce animal numbers. PO groups were fasted 4 h pre-dose. Blood (~0.3 mL, lithium heparin) was drawn at 0.167–24 h (PO) or 0.05–24 h (IV), centrifuged (2,500 × g, 15 min, 4 °C) within 1 h and plasma stored at ≤ −70 °C. At 24 h, femurs were excised, rinsed, blotted, weighed, stored at ≤ −70 °C and homogenised in 1 mL cold PBS pH 7.4. Primary outcome: plasma parent concentration over 24 h; secondary: femur concentration and femur/plasma ratio. Cage-side inspection accompanied every blood collection.

PK study 2—plasma and organs (rats, n = 6 per group). Doses of 2 mg/kg IV (pre-formulation diluted to 0.4 mg/mL in 0.9 % NaCl, 5 mL/kg) and 100 mg/kg PO (10 mg/mL pre-formulation undiluted, 10 mL/kg) were selected from the tolerability results; animals were unfasted. Tail-vein blood was sampled at 0.05, 0.167, 0.5 and 1 h and processed as above. At 1 h, animals were euthanised by CO_2 and liver, kidney, brain and thigh muscle were excised, rinsed, weighed and homogenised in cold PBS pH 7.4 (1.0 mL for kidney and brain, 10.0 mL for liver and muscle). Primary outcome: plasma parent concentration at 1 h; secondary: organ concentrations and organ/plasma ratios at 1 h, with cage-side inspection at each sampling.

Bioanalysis and data handling. As these were exploratory exposure and tolerability studies rather than hypothesis-testing experiments, no a priori sample size calculation, randomisation or blinding was applied, and no vehicle control group was included in the rat studieRat samples were processed by acetonitrile precipitation with oxybutynin as internal standard and analysed by LC–MS/MS (Poroshell 120 EC-C18, 2.7 µm, 3.0 × 50 mm, 40 °C; positive ESI-MRM m/z 278.1 → 96.1 and 358.3 → 142.3; SCIEX Triple Quad 6500^+ or 5500^+; $1/x^2$ weighted linear regression, 2–5000 ng/mL). LLOQ was 2 ng/mL (plasma) or 2 ng/g (tissue); values below 75 % of LLOQ (1.5 ng/mL or ng/g) were reported as BLOQ. Data are descriptive: body and organ weights as mean ± SEM and concentrations as mean ± SD, with individual values tabulated. Because most concentrations were BLOQ, no non-compartmental parameters (C_{max}, T_{max}, AUC, t½, CL, V_d, F) or tissue/plasma ratios could be derived; no inferential testing, effect sizes or confidence intervals are reported, and BLOQ values were not imputed.

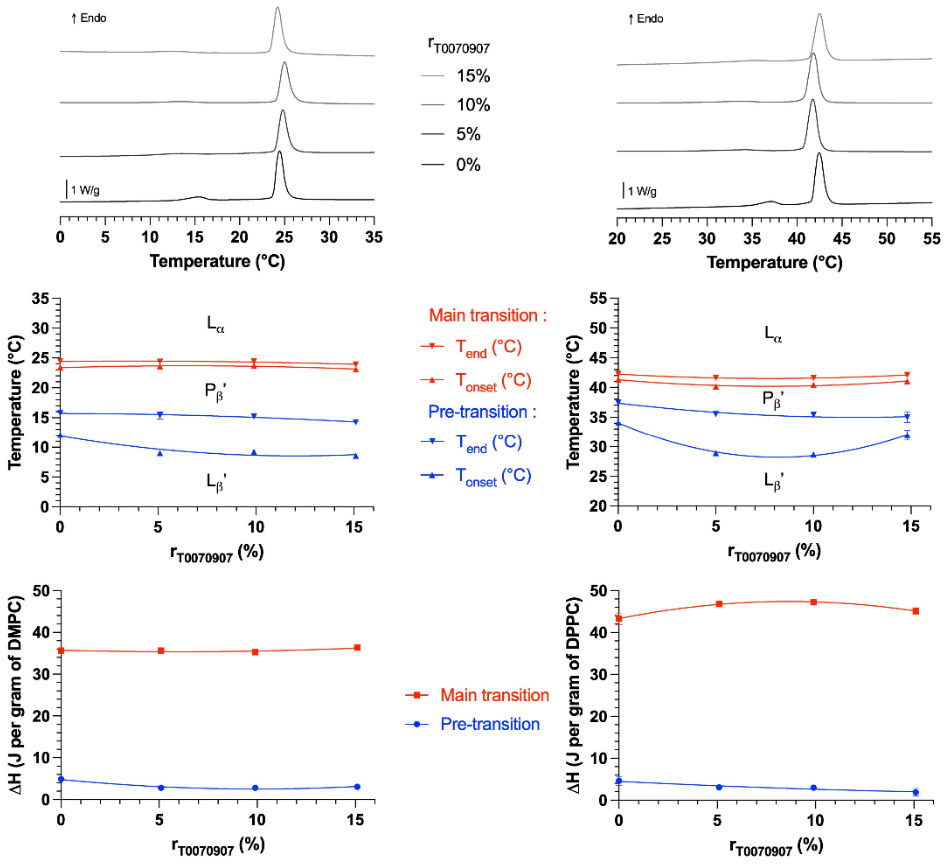

Supplementary Figure 1: Effect of T0070907 on the thermotropic behaviour of DMPC (left) and DPPC (right) bilayers assessed by differential scanning calorimetry.

(A, B) Representative DSC thermograms (heating scans, endothermic up) of DMPC (A) and DPPC (B) [multilamellar vesicles] containing increasing T0070907-to-lipid ratios ($r_{T0070907}$ = 0, 5, 10 and 15% [mol/mol]).

(C, D) Onset (T_{onset}, ▲) and completion (T_{end}, ▼) temperatures of the pre-transition (blue; $L_{\beta'} \rightarrow P_{\beta'}$) and main transition (red; $P_{\beta'} \rightarrow L_{\alpha}$) as a function of $r_{T0070907}$ for DMPC (C) and DPPC (D). The $L_{\beta'}$ (gel), $P_{\beta'}$ (ripple) and L_{α} (fluid) phase domains are indicated. **(E, F)** Enthalpy changes (ΔH, J per gram of phospholipid) of the pre-transition (blue ●) and main transition (red ■) as a function of $r_{T0070907}$ for DMPC (E) and DPPC (F).

Supplementary Fig. 2: AMES results.

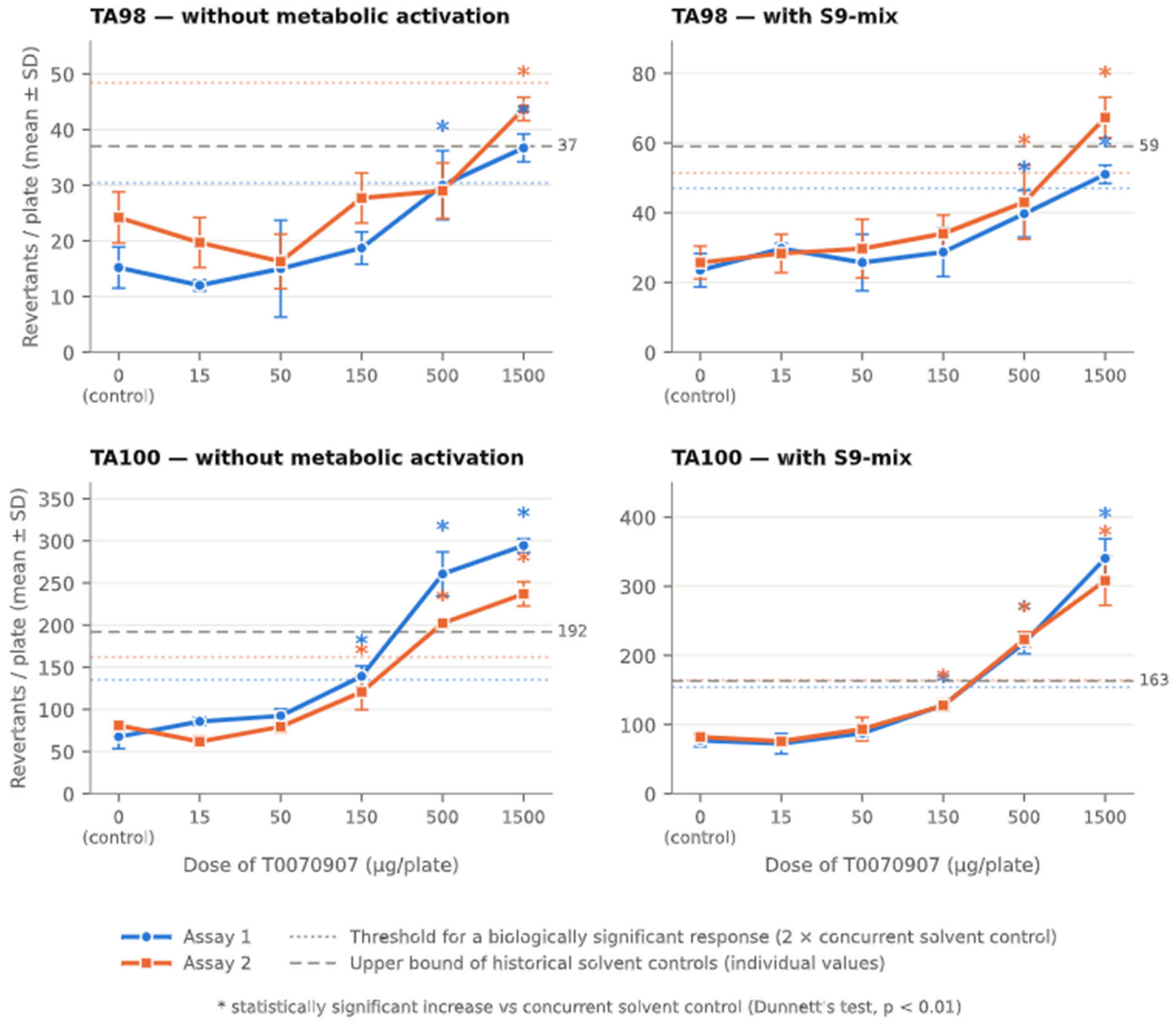

Supplementary Table 1: AMES raw data.

Strain	Assay	Activation	Protocol	Treatment	Dose (µg/plate)	Plate 1	Plate 2	Plate 3	Plate 4	Plate 5	Plate 6	n	Mean (recomputed)	SD (recomputed)	IR (recomputed)	Mean (report)	SD (report)	IR (report)	Dunnett's t	p
TA1535	1	Without S9-mix	Direct plate incorporation, without metabolic activation	**Solvent control (DMF)**	0	11	8	15	17	11	4	6	11,0	4,7	—	11	4,7	—	—	—
TA1535	1	Without S9-mix	Direct plate incorporation, without metabolic activation	**Positive control: Sodium azide (1 µg/plate)**	—	302	296	316				3	304,7	10,3	27,7	304,7	10,3	27,7	61,36	p<0.01
TA1535	1	Without S9-mix	Direct plate incorporation, without metabolic activation	Test item	15	8	4	4				3	5,3	2,3	0,5	5,3	2,3	0,5	2,57	N.S.
TA1535	1	Without S9-mix	Direct plate incorporation, without metabolic activation	Test item	50	9	10	8				3	9,0	1,0	0,8	9	1	0,8	0,91	N.S.
TA1535	1	Without S9-mix	Direct plate incorporation, without metabolic activation	Test item	150	13	10	10				3	11,0	1,7	1,0	11	1,7	1	0	N.S.
TA1535	1	Without S9-mix	Direct plate incorporation, without metabolic activation	Test item	500	8	10	5				3	7,7	2,5	0,7	7,7	2,5	0,7	1,51	N.S.
TA1535	1	Without S9-mix	Direct plate incorporation, without metabolic activation	Test item	1500	7	9	10				3	8,7	1,5	0,8	8,7	1,5	0,8	1,06	N.S.
TA1535	1	With S9-mix	Direct plate incorporation, with S9-mix, without pre-incubation	**Solvent control (DMF)**	0	12	8	10	5	12	21	6	11,3	5,4	—	11,3	5,4	—	—	—
TA1535	1	With S9-mix	Direct plate incorporation, with S9-mix, without pre-incubation	**Positive control: 2-anthramine (2 µg/plate)**	—	250	184	226				3	220,0	33,4	19,4	220	33,4	19,4	16,01	p<0.01
TA1535	1	With S9-mix	Direct plate incorporation, with S9-mix, without pre-incubation	Test item	15	7	6	5				3	6,0	1,0	0,5	6	1	0,5	2,2	N.S.
TA1535	1	With S9-mix	Direct plate incorporation, with S9-mix, without pre-incubation	Test item	50	13	10	13				3	12,0	1,7	1,1	12	1,7	1,1	0,27	N.S.
TA1535	1	With S9-mix	Direct plate incorporation, with S9-mix, without pre-incubation	Test item	150	8	7	7				3	7,3	0,6	0,6	7,3	0,6	0,6	1,65	N.S.
TA1535	1	With S9-mix	Direct plate incorporation, with S9-mix, without pre-incubation	Test item	500	7	7	9				3	7,7	1,2	0,7	7,7	1,2	0,7	1,51	N.S.
TA1535	1	With S9-mix	Direct plate incorporation, with S9-mix, without pre-incubation	Test item	1500	13	7	10				3	10,0	3,0	0,9	10	3	0,9	0,55	N.S.
TA1535	2	Without S9-mix	Direct plate incorporation, without metabolic activation (72 h incu	**Solvent control (DMF)**	0	16	6	9	2	10	8	6	8,5	4,6	—	8,5	4,6	—	—	—
TA1535	2	Without S9-mix	Direct plate incorporation, without metabolic activation (72 h incu	**Positive control: Sodium azide (1 µg/plate)**	—	432	384	376				3	397,3	30,3	46,7	397,3	30,3	46,7	33,01	p<0.01
TA1535	2	Without S9-mix	Direct plate incorporation, without metabolic activation (72 h incu	Test item	15	6	10	4				3	6,7	3,1	0,8	6,7	3,1	0,8	0,57	N.S.
TA1535	2	Without S9-mix	Direct plate incorporation, without metabolic activation (72 h incu	Test item	50	6	12	12				3	10,0	3,5	1,2	10	3,5	1,2	0,46	N.S.
TA1535	2	Without S9-mix	Direct plate incorporation, without metabolic activation (72 h incu	Test item	150	6	14	6				3	8,7	4,6	1,0	8,7	4,6	1	0,05	N.S.
TA1535	2	Without S9-mix	Direct plate incorporation, without metabolic activation (72 h incu	Test item	500	4	7	10				3	7,0	3,0	0,8	7	3	0,8	0,46	N.S.
TA1535	2	Without S9-mix	Direct plate incorporation, without metabolic activation (72 h incu	Test item	1500	17	5	18				3	13,3	7,2	1,6	13,3	7,2	1,6	1,49	N.S.
TA1535	2	With S9-mix	PRE-INCUBATION method (60 min at ca. 37 °C) with S9-mix; 72	**Solvent control (DMF)**	0	9	10	9	7	11	11	6	9,5	1,5	—	9,5	1,5	—	—	—
TA1535	2	With S9-mix	PRE-INCUBATION method (60 min at ca. 37 °C) with S9-mix; 72	**Positive control: 2-anthramine (1 µg/plate)**	—	238	202	198				3	212,7	22,0	22,4	212,7	22	22,4	24,26	p<0.01
TA1535	2	With S9-mix	PRE-INCUBATION method (60 min at ca. 37 °C) with S9-mix; 72	Test item	15	7	15	9				3	10,3	4,2	1,1	10,3	4,2	1,1	0,42	N.S.
TA1535	2	With S9-mix	PRE-INCUBATION method (60 min at ca. 37 °C) with S9-mix; 72	Test item	50	15	14	14				3	14,3	0,6	1,5	14,3	0,6	1,5	2,44	N.S.
TA1535	2	With S9-mix	PRE-INCUBATION method (60 min at ca. 37 °C) with S9-mix; 72	Test item	150	11	12	16				3	13,0	2,6	1,4	13	2,6	1,4	1,76	N.S.
TA1535	2	With S9-mix	PRE-INCUBATION method (60 min at ca. 37 °C) with S9-mix; 72	Test item	500	10	15	10				3	11,7	2,9	1,2	11,7	2,9	1,2	1,09	N.S.
TA1535	2	With S9-mix	PRE-INCUBATION method (60 min at ca. 37 °C) with S9-mix; 72	Test item	1500	9	13	18				3	13,3	4,5	1,4	13,3	4,5	1,4	1,93	N.S.
TA1537	1	Without S9-mix	Direct plate incorporation, without metabolic activation	**Solvent control (DMF)**	0	9	5	9	4	7	3	6	6,2	2,6	—	6,2	2,6	—	—	—
TA1537	1	Without S9-mix	Direct plate incorporation, without metabolic activation	**Positive control: 9-aminoacridine (50 µg/p**	—	320	410	452				3	394,0	67,4	63,9	394	67,4	63,9	15,19	p<0.01
TA1537	1	Without S9-mix	Direct plate incorporation, without metabolic activation	Test item	15	6	5	4				3	5,0	1,0	0,8	5	1	0,8	0,68	N.S.
TA1537	1	Without S9-mix	Direct plate incorporation, without metabolic activation	Test item	50	5	7	7				3	6,3	1,2	1,0	6,3	1,2	1	0,1	N.S.
TA1537	1	Without S9-mix	Direct plate incorporation, without metabolic activation	Test item	150	11	4	4				3	6,3	4,0	1,0	6,3	4	1	0,1	N.S.
TA1537	1	Without S9-mix	Direct plate incorporation, without metabolic activation	Test item	500	5	8	6				3	6,3	1,5	1,0	6,3	1,5	1	0,1	N.S.
TA1537	1	Without S9-mix	Direct plate incorporation, without metabolic activation	Test item	1500	8	5	10				3	7,7	2,5	1,2	7,7	2,5	1,2	0,88	N.S.
TA1537	1	With S9-mix	Direct plate incorporation, with S9-mix, without pre-incubation	**Solvent control (DMF)**	0	6	5	11	11	8	7	6	8,0	2,5	—	8	2,5	—	—	—
TA1537	1	With S9-mix	Direct plate incorporation, with S9-mix, without pre-incubation	**Positive control: 2-anthramine (2 µg/plate)**	—	124	109	84				3	105,7	20,2	13,2	105,7	20,2	13,2	12,54	p<0.01
TA1537	1	With S9-mix	Direct plate incorporation, with S9-mix, without pre-incubation	Test item	15	9	3	11				3	7,7	4,2	1,0	7,7	4,2	1	0,13	N.S.
TA1537	1	With S9-mix	Direct plate incorporation, with S9-mix, without pre-incubation	Test item	50	7	10	8				3	8,3	1,5	1,0	8,3	1,5	1	0,13	N.S.
TA1537	1	With S9-mix	Direct plate incorporation, with S9-mix, without pre-incubation	Test item	150	13	11	8				3	10,7	2,5	1,3	10,7	2,5	1,3	1,05	N.S.
TA1537	1	With S9-mix	Direct plate incorporation, with S9-mix, without pre-incubation	Test item	500	6	13	11				3	10,0	3,6	1,3	10	3,6	1,3	0,79	N.S.
TA1537	1	With S9-mix	Direct plate incorporation, with S9-mix, without pre-incubation	Test item	1500	17	4	11				3	10,7	6,5	1,3	10,7	6,5	1,3	1,05	N.S.
TA1537	2	Without S9-mix	Direct plate incorporation, without metabolic activation (72 h incu	**Solvent control (DMF)**	0	10	10	17	10	14	10	6	11,8	3,0	—	11,8	3	—	—	—
TA1537	2	Without S9-mix	Direct plate incorporation, without metabolic activation (72 h incu	**Positive control: 9-aminoacridine (50 µg/p**	—	186	252	202				3	213,3	34,4	18,0	213,3	34,4	18	15,34	p<0.01
TA1537	2	Without S9-mix	Direct plate incorporation, without metabolic activation (72 h incu	Test item	15	9	11	6				3	8,7	2,5	0,7	8,7	2,5	0,7	1,77	N.S.
TA1537	2	Without S9-mix	Direct plate incorporation, without metabolic activation (72 h incu	Test item	50	6	12	8				3	8,7	3,1	0,7	8,7	3,1	0,7	1,77	N.S.
TA1537	2	Without S9-mix	Direct plate incorporation, without metabolic activation (72 h incu	Test item	150	10	6	9				3	8,3	2,1	0,7	8,3	2,1	0,7	1,96	N.S.
TA1537	2	Without S9-mix	Direct plate incorporation, without metabolic activation (72 h incu	Test item	500	5	8	6				3	6,3	1,5	0,5	6,3	1,5	0,5	3,08	N.S.
TA1537	2	Without S9-mix	Direct plate incorporation, without metabolic activation (72 h incu	Test item	1500	7	10	10				3	9,0	1,7	0,8	9	1,7	0,8	1,59	N.S.
TA1537	2	With S9-mix	PRE-INCUBATION method (60 min at ca. 37 °C) with S9-mix; 72	**Solvent control (DMF)**	0	9	13	5	17	14	10	6	11,3	4,2	—	11,3	4,2	—	—	—
TA1537	2	With S9-mix	PRE-INCUBATION method (60 min at ca. 37 °C) with S9-mix; 72	**Positive control: 2-anthramine (1 µg/plate)**	—	104	122	72				3	99,3	25,3	8,8	99,3	25,3	8,8	8,89	p<0.01
TA1537	2	With S9-mix	PRE-INCUBATION method (60 min at ca. 37 °C) with S9-mix; 72	Test item	15	13	7	7				3	9,0	3,5	0,8	9	3,5	0,8	0,81	N.S.
TA1537	2	With S9-mix	PRE-INCUBATION method (60 min at ca. 37 °C) with S9-mix; 72	Test item	50	12	11	8				3	10,3	2,1	0,9	10,3	2,1	0,9	0,35	N.S.
TA1537	2	With S9-mix	PRE-INCUBATION method (60 min at ca. 37 °C) with S9-mix; 72	Test item	150	18	8	17				3	14,3	5,5	1,3	14,3	5,5	1,3	1,05	N.S.
TA1537	2	With S9-mix	PRE-INCUBATION method (60 min at ca. 37 °C) with S9-mix; 72	Test item	500	15	14	8				3	12,3	3,8	1,1	12,3	3,8	1,1	0,35	N.S.
TA1537	2	With S9-mix	PRE-INCUBATION method (60 min at ca. 37 °C) with S9-mix; 72	Test item	1500	12	18	10				3	13,3	4,2	1,2	13,3	4,2	1,2	0,7	N.S.
TA98	1	Without S9-mix	Direct plate incorporation, without metabolic activation	**Solvent control (DMF)**	0	13	12	15	12	18	21	6	15,2	3,7	—	15,2	3,7	—	—	—
TA98	1	Without S9-mix	Direct plate incorporation, without metabolic activation	**Positive control: 2-nitrofluorene (2 µg/plat**	—	336	270	332				3	312,7	37,0	20,6	312,7	37	20,6	21,02	p<0.01
TA98	1	Without S9-mix	Direct plate incorporation, without metabolic activation	Test item	15	13	12	11				3	12,0	1,0	0,8	12	1	0,8	0,96	N.S.
TA98	1	Without S9-mix	Direct plate incorporation, without metabolic activation	Test item	50	25	10	10				3	15,0	8,7	1,0	15	8,7	1	0,05	N.S.
TA98	1	Without S9-mix	Direct plate incorporation, without metabolic activation	Test item	150	22	17	17				3	18,7	2,9	1,2	18,7	2,9	1,2	1,06	N.S.
TA98	1	Without S9-mix	Direct plate incorporation, without metabolic activation	Test item	500	35	32	23				3	30,0	6,2	2,0	30	6,2	2	4,5	p<0.01
TA98	1	Without S9-mix	Direct plate incorporation, without metabolic activation	Test item	1500	39	34	37				3	36,7	2,5	2,4	36,7	2,5	2,4	6,52	p<0.01
TA98	1	With S9-mix	Direct plate incorporation, with S9-mix, without pre-incubation	**Solvent control (DMF)**	0	26	27	17	18	25	28	6	23,5	4,8	—	23,5	4,8	—	—	—
TA98	1	With S9-mix	Direct plate incorporation, with S9-mix, without pre-incubation	**Positive control: 2-anthramine (2 µg/plate)**	—	1628	1576	1816				3	1673,3	126,3	71,2	1673,3	126,3	71,2	34,51	p<0.01
TA98	1	With S9-mix	Direct plate incorporation, with S9-mix, without pre-incubation	Test item	15	30	31	28				3	29,7	1,5	1,3	29,7	1,5	1,3	1,59	N.S.
TA98	1	With S9-mix	Direct plate incorporation, with S9-mix, without pre-incubation	Test item	50	35	22	20				3	25,7	8,1	1,1	25,7	8,1	1,1	0,56	N.S.
TA98	1	With S9-mix	Direct plate incorporation, with S9-mix, without pre-incubation	Test item	150	28	22	36				3	28,7	7,0	1,2	28,7	7	1,2	1,33	N.S.
TA98	1	With S9-mix	Direct plate incorporation, with S9-mix, without pre-incubation	Test item	500	38	34	47				3	39,7	6,7	1,7	39,7	6,7	1,7	4,16	p<0.01
TA98	1	With S9-mix	Direct plate incorporation, with S9-mix, without pre-incubation	Test item	1500	52	53	48				3	51,0	2,6	2,2	51	2,6	2,2	7,08	p<0.01
TA98	2	Without S9-mix	Direct plate incorporation, without metabolic activation (72 h incu	**Solvent control (DMF)**	0	23	28	31	19	20	24	6	24,2	4,6	—	24,2	4,6	—	—	—
TA98	2	Without S9-mix	Direct plate incorporation, without metabolic activation (72 h incu	**Positive control: 2-nitrofluorene (2 µg/plat**	—	366	350	298				3	338,0	35,6	14,0	338	35,6	14	22,88	p<0.01
TA98	2	Without S9-mix	Direct plate incorporation, without metabolic activation (72 h incu	Test item	15	20	15	24				3	19,7	4,5	0,8	19,7	4,5	0,8	1,43	N.S.
TA98	2	Without S9-mix	Direct plate incorporation, without metabolic activation (72 h incu	Test item	50	13	14	22				3	16,3	4,9	0,7	16,3	4,9	0,7	2,5	N.S.
TA98	2	Without S9-mix	Direct plate incorporation, without metabolic activation (72 h incu	Test item	150	32	28	23				3	27,7	4,5	1,1	27,7	4,5	1,1	1,12	N.S.
TA98	2	Without S9-mix	Direct plate incorporation, without metabolic activation (72 h incu	Test item	500	29	34	24				3	29,0	5,0	1,2	29	5	1,2	1,54	N.S.
TA98	2	Without S9-mix	Direct plate incorporation, without metabolic activation (72 h incu	Test item	1500	43	46	42				3	43,7	2,1	1,8	43,7	2,1	1,8	6,21	p<0.01
TA98	2	With S9-mix	Direct plate incorporation with S9-mix, WITHOUT pre-incubation	**Solvent control (DMF)**	0	24	23	34	28	24	21	6	25,7	4,7	—	25,7	4,7	—	—	—
TA98	2	With S9-mix	Direct plate incorporation with S9-mix, WITHOUT pre-incubation	**Positive control: 2-anthramine (2 µg/plate)**	—	1152	1368	1000				3	1173,3	184,9	45,7	1173,3	184,9	45,7	16,41	p<0.01
TA98	2	With S9-mix	Direct plate incorporation with S9-mix, WITHOUT pre-incubation	Test item	15	28	23	34				3	28,3	5,5	1,1	28,3	5,5	1,1	0,57	N.S.
TA98	2	With S9-mix	Direct plate incorporation with S9-mix, WITHOUT pre-incubation	Test item	50	35	20	34				3	29,7	8,4	1,2	29,7	8,4	1,2	0,85	N.S.
TA98	2	With S9-mix	Direct plate incorporation with S9-mix, WITHOUT pre-incubation	Test item	150	36	28	38				3	34,0	5,3	1,3	34	5,3	1,3	1,78	N.S.
TA98	2	With S9-mix	Direct plate incorporation with S9-mix, WITHOUT pre-incubation	Test item	500	55	35	39				3	43,0	10,6	1,7	43	10,6	1,7	3,7	p<0.01
TA98	2	With S9-mix	Direct plate incorporation with S9-mix, WITHOUT pre-incubation	Test item	1500	64	74	64				3	67,3	5,8	2,6	67,3	5,8	2,6	8,9	p<0.01

TA100	1	Without S9-mix	Direct plate incorporation, without metabolic activation	**Solvent control (DMF)**	0	61	73	46	73	88	64	6	67,5	14,1	—	67,5	14,1	—	—	—
TA100	1	Without S9-mix	Direct plate incorporation, without metabolic activation	**Positive control: Sodium azide (1 µg/plate)**	—	688	636	576				3	633,3	56,0	9,4	633,3	56	9,4	24,81	p<0.01
TA100	1	Without S9-mix	Direct plate incorporation, without metabolic activation	Test item	15	88	89	80				3	85,7	4,9	1,3	85,7	4,9	1,3	1,82	N.S.
TA100	1	Without S9-mix	Direct plate incorporation, without metabolic activation	Test item	50	95	99	83				3	92,3	8,3	1,4	92,3	8,3	1,4	2,49	N.S.
TA100	1	Without S9-mix	Direct plate incorporation, without metabolic activation	Test item	150	150	126	142				3	139,3	12,2	2,1	139,3	12,2	2,1	7,21	p<0.01
TA100	1	Without S9-mix	Direct plate incorporation, without metabolic activation	Test item	500	288	258	236				3	260,7	26,1	3,9	260,7	26,1	3,9	19,39	p<0.01
TA100	1	Without S9-mix	Direct plate incorporation, without metabolic activation	Test item	1500	298	300	285				3	294,3	8,1	4,4	294,3	8,1	4,4	22,76	p<0.01
TA100	1	With S9-mix	Direct plate incorporation, with S9-mix, without pre-incubation	**Solvent control (DMF)**	0	85	66	79	69	74	89	6	77,0	9,0	—	77	9	—	—	—
TA100	1	With S9-mix	Direct plate incorporation, with S9-mix, without pre-incubation	**Positive control: 2-anthramine (2 µg/plate)**	—	1256	1296	1160				3	1237,3	69,9	16,1	1237,3	69,9	16,1	43,04	p<0.01
TA100	1	With S9-mix	Direct plate incorporation, with S9-mix, without pre-incubation	Test item	15	61	67	89				3	72,3	14,7	0,9	72,3	14,7	0,9	0,47	N.S.
TA100	1	With S9-mix	Direct plate incorporation, with S9-mix, without pre-incubation	Test item	50	86	93	83				3	87,3	5,1	1,1	87,3	5,1	1,1	1,03	N.S.
TA100	1	With S9-mix	Direct plate incorporation, with S9-mix, without pre-incubation	Test item	150	127	131	126				3	128,0	2,6	1,7	128	2,6	1,7	5,1	p<0.01
TA100	1	With S9-mix	Direct plate incorporation, with S9-mix, without pre-incubation	Test item	500	236	208	210				3	218,0	15,6	2,8	218	15,6	2,8	14,11	p<0.01
TA100	1	With S9-mix	Direct plate incorporation, with S9-mix, without pre-incubation	Test item	1500	348	364	309				3	340,3	28,3	4,4	340,3	28,3	4,4	26,35	p<0.01
TA100	2	Without S9-mix	Direct plate incorporation, without metabolic activation (72 h incu	**Solvent control (DMF)**	0	84	91	79	72	83	77	6	81,0	6,5	—	81	6,5	—	—	—
TA100	2	Without S9-mix	Direct plate incorporation, without metabolic activation (72 h incu	**Positive control: Sodium azide (1 µg/plate)**	—	800	728	720				3	749,3	44,1	9,3	749,3	44,1	9,3	39,07	p<0.01
TA100	2	Without S9-mix	Direct plate incorporation, without metabolic activation (72 h incu	Test item	15	56	69	60				3	61,7	6,7	0,8	61,7	6,7	0,8	2,55	N.S.
TA100	2	Without S9-mix	Direct plate incorporation, without metabolic activation (72 h incu	Test item	50	72	80	86				3	79,3	7,0	1,0	79,3	7	1	0,22	N.S.
TA100	2	Without S9-mix	Direct plate incorporation, without metabolic activation (72 h incu	Test item	150	108	109	145				3	120,7	21,1	1,5	120,7	21,1	1,5	5,23	p<0.01
TA100	2	Without S9-mix	Direct plate incorporation, without metabolic activation (72 h incu	Test item	500	201	200	206				3	202,3	3,2	2,5	202,3	3,2	2,5	15,99	p<0.01
TA100	2	Without S9-mix	Direct plate incorporation, without metabolic activation (72 h incu	Test item	1500	253	233	225				3	237,0	14,4	2,9	237	14,4	2,9	20,56	p<0.01
TA100	2	With S9-mix	Direct plate incorporation with S9-mix, WITHOUT pre-incubation	**Solvent control (DMF)**	0	79	82	82	80	89	80	6	82,0	3,6	—	82	3,6	—	—	—
TA100	2	With S9-mix	Direct plate incorporation with S9-mix, WITHOUT pre-incubation	**Positive control: 2-anthramine (2 µg/plate)**	—	920	1280	1120				3	1106,7	180,4	13,5	1106,7	180,4	13,5	15,02	p<0.01
TA100	2	With S9-mix	Direct plate incorporation with S9-mix, WITHOUT pre-incubation	Test item	15	80	76	71				3	75,7	4,5	0,9	75,7	4,5	0,9	0,58	N.S.
TA100	2	With S9-mix	Direct plate incorporation with S9-mix, WITHOUT pre-incubation	Test item	50	76	94	110				3	93,3	17,0	1,1	93,3	17	1,1	1,03	N.S.
TA100	2	With S9-mix	Direct plate incorporation with S9-mix, WITHOUT pre-incubation	Test item	150	136	128	119				3	127,7	8,5	1,6	127,7	8,5	1,6	4,15	p<0.01
TA100	2	With S9-mix	Direct plate incorporation with S9-mix, WITHOUT pre-incubation	Test item	500	221	213	235				3	223,0	11,1	2,7	223	11,1	2,7	12,83	p<0.01
TA100	2	With S9-mix	Direct plate incorporation with S9-mix, WITHOUT pre-incubation	Test item	1500	349	291	284				3	308,0	35,7	3,8	308	35,7	3,8	20,56	p<0.01

TA102	1	Without S9-mix	Direct plate incorporation, without metabolic activation	**Solvent control (DMF)**	0	168	150	224	165	220	224	6	191,8	34,4	—	191,8	34,4	—	—	—
TA102	1	Without S9-mix	Direct plate incorporation, without metabolic activation	**Positive control: Mitomycin C (0.125 µg/pla**	—	1296	1424	1048				3	1256,0	191,2	6,5	1256	191,2	6,5	14,17	p<0.01
TA102	1	Without S9-mix	Direct plate incorporation, without metabolic activation	Test item	15	137	177	189				3	167,7	27,2	0,9	167,7	27,2	0,9	1,19	N.S.
TA102	1	Without S9-mix	Direct plate incorporation, without metabolic activation	Test item	50	209	153	185				3	182,3	28,1	1,0	182,3	28,1	1	0,47	N.S.
TA102	1	Without S9-mix	Direct plate incorporation, without metabolic activation	Test item	150	179	171	211				3	187,0	21,2	1,0	187	21,2	1	0,24	N.S.
TA102	1	Without S9-mix	Direct plate incorporation, without metabolic activation	Test item	500	183	159	133				3	158,3	25,0	0,8	158,3	25	0,8	1,65	N.S.
TA102	1	Without S9-mix	Direct plate incorporation, without metabolic activation	Test item	1500	125	124	81				3	110,0	25,1	0,6	110	25,1	0,6	4,03	p<0.01
TA102	1	With S9-mix	Direct plate incorporation, with S9-mix, without pre-incubation	**Solvent control (DMF)**	0	224	208	272	286	318	292	6	266,7	42,3	—	266,7	42,3	—	—	—
TA102	1	With S9-mix	Direct plate incorporation, with S9-mix, without pre-incubation	**Positive control: Benzo[a]pyrene (2 µg/pla**	—	2608	2336	2368				3	2437,3	148,7	9,1	2437,3	148,7	9,1	35,23	p<0.01
TA102	1	With S9-mix	Direct plate incorporation, with S9-mix, without pre-incubation	Test item	15	258	306	342				3	302,0	42,1	1,1	302	42,1	1,1	0,86	N.S.
TA102	1	With S9-mix	Direct plate incorporation, with S9-mix, without pre-incubation	Test item	50	220	284	326				3	276,7	53,4	1,0	276,7	53,4	1	0,24	N.S.
TA102	1	With S9-mix	Direct plate incorporation, with S9-mix, without pre-incubation	Test item	150	380	380	352				3	370,7	16,2	1,4	370,7	16,2	1,4	2,53	N.S.
TA102	1	With S9-mix	Direct plate incorporation, with S9-mix, without pre-incubation	Test item	500	324	304	354				3	327,3	25,2	1,2	327,3	25,2	1,2	1,47	N.S.
TA102	1	With S9-mix	Direct plate incorporation, with S9-mix, without pre-incubation	Test item	1500	326	290	95				3	237,0	124,3	0,9	237	124,3	0,9	0,72	N.S.
TA102	2	Without S9-mix	Direct plate incorporation, without metabolic activation (72 h incu	**Solvent control (DMF)**	0	228	236	240	242	272	264	6	247,0	17,1	—	247	17,1	—	—	—
TA102	2	Without S9-mix	Direct plate incorporation, without metabolic activation (72 h incu	**Positive control: Mitomycin C (0.125 µg/pla**	—	1584	1616	1696				3	1632,0	57,7	6,6	1632	57,7	6,6	57,49	p<0.01
TA102	2	Without S9-mix	Direct plate incorporation, without metabolic activation (72 h incu	Test item	15	162	166	208				3	178,7	25,5	0,7	178,7	25,5	0,7	4,02	p<0.01
TA102	2	Without S9-mix	Direct plate incorporation, without metabolic activation (72 h incu	Test item	50	246	210	222				3	226,0	18,3	0,9	226	18,3	0,9	1,24	N.S.
TA102	2	Without S9-mix	Direct plate incorporation, without metabolic activation (72 h incu	Test item	150	258	222	242				3	240,7	18,0	1,0	240,7	18	1	0,37	N.S.
TA102	2	Without S9-mix	Direct plate incorporation, without metabolic activation (72 h incu	Test item	500	236	166	240				3	214,0	41,6	0,9	214	41,6	0,9	1,94	N.S.
TA102	2	Without S9-mix	Direct plate incorporation, without metabolic activation (72 h incu	Test item	1500	194	230	186				3	203,3	23,4	0,8	203,3	23,4	0,8	2,57	N.S.
TA102	2	With S9-mix	PRE-INCUBATION method (60 min at ca. 37 °C) with S9-mix; 72	**Solvent control (DMF)**	0	272	278	226	314	282	264	6	272,7	28,6	—	272,7	28,6	—	—	—
TA102	2	With S9-mix	PRE-INCUBATION method (60 min at ca. 37 °C) with S9-mix; 72	**Positive control: Benzo[a]pyrene (2 µg/pla**	—	1032	840	824				3	898,7	115,7	3,3	898,7	115,7	3,3	13,33	p<0.01
TA102	2	With S9-mix	PRE-INCUBATION method (60 min at ca. 37 °C) with S9-mix; 72	Test item	15	272	218	284				3	258,0	35,2	0,9	258	35,2	0,9	0,45	N.S.
TA102	2	With S9-mix	PRE-INCUBATION method (60 min at ca. 37 °C) with S9-mix; 72	Test item	50	360	306	266				3	310,7	47,2	1,1	310,7	47,2	1,1	1,18	N.S.
TA102	2	With S9-mix	PRE-INCUBATION method (60 min at ca. 37 °C) with S9-mix; 72	Test item	150	292	336	342				3	323,3	27,3	1,2	323,3	27,3	1,2	1,57	N.S.
TA102	2	With S9-mix	PRE-INCUBATION method (60 min at ca. 37 °C) with S9-mix; 72	Test item	500	352	388	252				3	330,7	70,5	1,2	330,7	70,5	1,2	1,8	N.S.
TA102	2	With S9-mix	PRE-INCUBATION method (60 min at ca. 37 °C) with S9-mix; 72	Test item	1500	252	202	120				3	191,3	66,6	0,7	191,3	66,6	0,7	2,52	N.S.

Strain	Activation	Assay	Dose (µg/plate)	Plate 1	Plate 2	Plate 3	Mean	SD	IR	Biological threshold (2 × control)	≥ threshold?	Historical upper bound	> historical bound?	Dunnett's t	p
TA98	Without S9-mix	1	**0 (solvent control)**	13	12	15	**15,2**	**3,7**	—	30,4	—	37	—	—	—
TA98	Without S9-mix	1	15	13	12	11	12,0	1,0	0,8	30,4	no	37	no	0,96	N.S.
TA98	Without S9-mix	1	50	25	10	10	15,0	8,7	1,0	30,4	no	37	no	0,05	N.S.
TA98	Without S9-mix	1	150	22	17	17	18,7	2,9	1,2	30,4	no	37	no	1,06	N.S.
TA98	Without S9-mix	1	500	35	32	23	30,0	6,2	2,0	30,4	no	37	no	4,5	p<0.01
TA98	Without S9-mix	1	1500	39	34	37	36,7	2,5	2,4	30,4	yes	37	no	6,52	p<0.01
TA98	Without S9-mix	2	**0 (solvent control)**	23	28	31	**24,2**	**4,6**	—	48,4	—	37	—	—	—
TA98	Without S9-mix	2	15	20	15	24	19,7	4,5	0,8	48,4	no	37	no	1,43	N.S.
TA98	Without S9-mix	2	50	13	14	22	16,3	4,9	0,7	48,4	no	37	no	2,5	N.S.
TA98	Without S9-mix	2	150	32	28	23	27,7	4,5	1,1	48,4	no	37	no	1,12	N.S.
TA98	Without S9-mix	2	500	29	34	24	29,0	5,0	1,2	48,4	no	37	no	1,54	N.S.
TA98	Without S9-mix	2	1500	43	46	42	43,7	2,1	1,8	48,4	no	37	yes	6,21	p<0.01
TA98	With S9-mix	1	**0 (solvent control)**	26	27	17	**23,5**	**4,8**	—	47,0	—	59	—	—	—
TA98	With S9-mix	1	15	30	31	28	29,7	1,5	1,3	47,0	no	59	no	1,59	N.S.
TA98	With S9-mix	1	50	35	22	20	25,7	8,1	1,1	47,0	no	59	no	0,56	N.S.
TA98	With S9-mix	1	150	28	22	36	28,7	7,0	1,2	47,0	no	59	no	1,33	N.S.
TA98	With S9-mix	1	500	38	34	47	39,7	6,7	1,7	47,0	no	59	no	4,16	p<0.01
TA98	With S9-mix	1	1500	52	53	48	51,0	2,6	2,2	47,0	yes	59	no	7,08	p<0.01
TA98	With S9-mix	2	**0 (solvent control)**	24	23	34	**25,7**	**4,7**	—	51,4	—	59	—	—	—
TA98	With S9-mix	2	15	28	23	34	28,3	5,5	1,1	51,4	no	59	no	0,57	N.S.
TA98	With S9-mix	2	50	35	20	34	29,7	8,4	1,2	51,4	no	59	no	0,85	N.S.
TA98	With S9-mix	2	150	36	28	38	34,0	5,3	1,3	51,4	no	59	no	1,78	N.S.
TA98	With S9-mix	2	500	55	35	39	43,0	10,6	1,7	51,4	no	59	no	3,7	p<0.01
TA98	With S9-mix	2	1500	64	74	64	67,3	5,8	2,6	51,4	yes	59	yes	8,9	p<0.01
TA100	Without S9-mix	1	**0 (solvent control)**	61	73	46	**67,5**	**14,1**	—	135,0	—	192	—	—	—
TA100	Without S9-mix	1	15	88	89	80	85,7	4,9	1,3	135,0	no	192	no	1,82	N.S.
TA100	Without S9-mix	1	50	95	99	83	92,3	8,3	1,4	135,0	no	192	no	2,49	N.S.
TA100	Without S9-mix	1	150	150	126	142	139,3	12,2	2,1	135,0	yes	192	no	7,21	p<0.01
TA100	Without S9-mix	1	500	288	258	236	260,7	26,1	3,9	135,0	yes	192	yes	19,39	p<0.01
TA100	Without S9-mix	1	1500	298	300	285	294,3	8,1	4,4	135,0	yes	192	yes	22,76	p<0.01
TA100	Without S9-mix	2	**0 (solvent control)**	84	91	79	**81,0**	**6,5**	—	162,0	—	192	—	—	—
TA100	Without S9-mix	2	15	56	69	60	61,7	6,7	0,8	162,0	no	192	no	2,55	N.S.
TA100	Without S9-mix	2	50	72	80	86	79,3	7,0	1,0	162,0	no	192	no	0,22	N.S.
TA100	Without S9-mix	2	150	108	109	145	120,7	21,1	1,5	162,0	no	192	no	5,23	p<0.01
TA100	Without S9-mix	2	500	201	200	206	202,3	3,2	2,5	162,0	yes	192	yes	15,99	p<0.01
TA100	Without S9-mix	2	1500	253	233	225	237,0	14,4	2,9	162,0	yes	192	yes	20,56	p<0.01
TA100	With S9-mix	1	**0 (solvent control)**	85	66	79	**77,0**	**9,0**	—	154,0	—	163	—	—	—
TA100	With S9-mix	1	15	61	67	89	72,3	14,7	0,9	154,0	no	163	no	0,47	N.S.
TA100	With S9-mix	1	50	86	93	83	87,3	5,1	1,1	154,0	no	163	no	1,03	N.S.
TA100	With S9-mix	1	150	127	131	126	128,0	2,6	1,7	154,0	no	163	no	5,1	p<0.01
TA100	With S9-mix	1	500	236	208	210	218,0	15,6	2,8	154,0	yes	163	yes	14,11	p<0.01
TA100	With S9-mix	1	1500	348	364	309	340,3	28,3	4,4	154,0	yes	163	yes	26,35	p<0.01
TA100	With S9-mix	2	**0 (solvent control)**	79	82	82	**82,0**	**3,6**	—	164,0	—	163	—	—	—
TA100	With S9-mix	2	15	80	76	71	75,7	4,5	0,9	164,0	no	163	no	0,58	N.S.
TA100	With S9-mix	2	50	76	94	110	93,3	17,0	1,1	164,0	no	163	no	1,03	N.S.
TA100	With S9-mix	2	150	136	128	119	127,7	8,5	1,6	164,0	no	163	no	4,15	p<0.01
TA100	With S9-mix	2	500	221	213	235	223,0	11,1	2,7	164,0	yes	163	yes	12,83	p<0.01
TA100	With S9-mix	2	1500	349	291	284	308,0	35,7	3,8	164,0	yes	163	yes	20,56	p<0.01
